# Social comparison and segregation reveal well-being traps

**DOI:** 10.1057/s41599-026-07875-9

**Published:** 2026-06-11

**Authors:** Eric Dignum, Arend Geerlofs, Lasse Gerrits, Debraj Roy

**Affiliations:** 1 University of Amsterdam, Amsterdam, The Netherlands; 2 Erasmus University Rotterdam, Rotterdam, The Netherlands

**Keywords:** Complex networks, Complex networks, Psychology, Psychology, Sociology

## Abstract

The Easterlin paradox suggests that while higher income correlates with greater subjective well-being (SWB) cross-sectionally, rising incomes over time fail to yield increased SWB. Traditional explanations, focused on social comparison and hedonic adaptation, often neglect individual variability and dynamic social structures. Using an agent-based model (ABM) that incorporates heterogeneity and social relationships, we uncover several key insights. We find that social comparison significantly hinders adaptation to economic shocks, with downward comparisons enhancing resilience and upward comparisons reducing long-term SWB while increasing instability. It also functions as a double-edged sword, amplifying long-term SWB changes during localised shocks, but buffering societal impacts when shocks are widespread. Furthermore, low-income individuals are particularly prone to low well-being traps, a vulnerability partially mitigated by segregated comparison groups. We show that social comparison’s dual nature demands context-specific policies. By mitigating upward comparison risks in localised crises and leveraging downward comparisons during widespread shocks, policymakers can enhance societal resilience while addressing systemic inequities.

## Introduction

The relationship between a person’s general economic situation and their subjective well-being (SWB)^1^ has been a subject of debate and research, often referred to as the “Easterlin paradox" (Easterlin, 1974). This paradox suggests that at a given point in time, individuals with higher incomes have higher SWB than those with lower incomes, both within and across countries. However, over time, as the average incomes increase in a country, the average levels of SWB do not increase correspondingly (Easterlin, 1974; Easterlin and O’Connor, 2022; Wolbring et al., 2013). This creates a paradox: income and SWB are positively correlated in cross-sectional data but not in long-term time series data.

At any point in time, more affluent people have higher SWB than average because most of the people with whom they compare themselves are worse off, and vice versa for the less affluent. This social comparison mechanism indicates that individuals derive utility not (only) from their absolute income, but from their income or rank relative to others (Boyce et al., 2010; Brown et al., 2008; Easterlin and O’Connor, 2022; Hopkins and Kornienko, 2009; Lakshmanasamy and Maya, 2020; Osafo Hounkpatin et al., 2015; Paul and Guilbert, 2013; Wolbring et al., 2013). However, when individual incomes increase, so do the reference levels, and hence this explains the absence of an effect over time. Additionally, through hedonic adaptation, people get used to higher (or lower) living standards, so the initial boosts in SWB from increased income diminish over time (Brickman, 1971; Diener et al., 2009). Importantly, hedonic adaptation can explain (part of) the time series component of the paradox, but social comparison is a necessary ingredient for the cross-sectional relationship (Easterlin and O’Connor, 2022).

Understanding the Easterlin paradox is important for policymaking for several reasons. First, it challenges the idea that economic growth alone leads to increased happiness and well-being. Second, the paradox suggests that an individual’s SWB is more influenced by their relative income compared to others, rather than absolute income levels. Finally, the paradox distinguishes between short-term fluctuations and long-term trends in SWB. This distinction is crucial for policy makers when designing policies and evaluating their impact across different time horizons. Additionally, research demonstrates important connections between SWB and susceptibility to misinformation and disinformation (Rauwolf, 2022), longevity (Diener et al., 2018), institutional trust and civic engagement (Tov and Diener, 2009). Understanding the mechanisms that shape SWB trajectories therefore has implications extending well beyond individual satisfaction to encompass public health, political stability, and social cohesion.

Although there has been substantial evidence for the validity of the Easterlin paradox (Boyce et al., 2010; Brown et al., 2008; Easterlin and O’Connor, 2022; Hopkins and Kornienko, 2009; Lakshmanasamy and Maya, 2020; Osafo Hounkpatin et al., 2015; Paul and Guilbert, 2013; Wolbring et al., 2013), it remains an aggregate empirical finding. However, heterogeneity is found to be an important predictor of variance in SWB (Diener et al., 2009) and within-country inequality with respect to life satisfaction has been increasing for more than a decade (Helliwell et al., 2025). Therefore, it is crucial to understand individual differences in SWB. For example, without disproving the paradox, for some individuals increases in income may indeed lead to long-term improvements in SWB (i.e., a positive association), while for others the opposite holds. Understanding such heterogeneity is important to identify vulnerable populations and design more targeted interventions, but it is less clear under which individual and societal conditions increases or decreases in individual income can lead to long-lasting changes in SWB. For example: How do individual and societal SWB evolve with changing wealth distributions and inequality? Moreover, individuals tend to interact preferentially with others who are similar to themselves (McPherson et al., 2001), including along economic dimensions. Thus, the people with whom we compare ourselves (that is, the comparison or reference group) likely have similar income levels. This raises questions about the impact of segregation, social comparison, and their effect on SWB within and between communities. Furthermore, we tend to make upward comparisons (e.g., to those who earn or have more), which negatively affects our SWB (Gerber et al., 2018), but what is the nature of the relationship between different levels of social comparison and SWB? Hence, both individual differences (e.g., heterogeneity) and social characteristics (e.g., segregation) are potentially important in identifying for which individuals increases or decreases in income could lead to long-lasting changes in SWB and why. In addition, although the Easterlin paradox is about long-term temporal changes in income and SWB, it is also valuable to take resilience and stability into account. These metrics are in line with the more dynamic measures of SWB (Galatzer-Levy et al., 2018; Schäfer et al., 2024) and are often overlooked. For example, how do social comparison and hedonic adaptation influence the resilience of individuals and communities in the context of shocks? Such insights are important in designing interventions beyond economic metrics alone and improving individual and community resilience (Atkinson et al., 2020).

However, models that explicitly take such heterogeneity, adaptation, and social structures (e.g., segregation, comparison groups) into account are largely unexplored. Existing studies (Baggio and Papyrakis, 2014; Bishop and Kavak, 2023; Bruno, 2025; Dubey et al., 2022; Schmalz et al., 2007; Silvestre et al., 2019; Thron, 2014) have focused mainly on individual aspects or highly aggregate levels, with limited integration of adaptation mechanisms, dynamic social factors, and interactions between them. This is partly driven by the fact that conventional statistical analyses often do not adequately capture emergent dynamics and feedback loops. However, social comparison, adaptation mechanisms, segregation, and inequality can interact in non-trivial ways. Such dynamics can contribute to the non-linear relationship between key factors and SWB, as changes on the societal/community level can feedback into the individual level, and vice versa (Vanags et al., 2025). These limitations and the need to test counterfactuals highlight the need for more comprehensive frameworks to capture the dynamics of SWB consistent with existing theories and empirical evidence. Although recent statistical analyses have identified associations between individual, social and place-based factors that influence SWB (Diener et al., 2018; Jasielska et al., 2021; McElroy et al., 2021; VanderWeele, 2017), a better understanding of the mechanisms shaped by complex interactions of these factors is lacking.

A notable methodology that can explicitly account for individual heterogeneous behaviours, dynamic social structures, and interactions between them, is agent-based modelling. Agent-based models (ABMs) aim to describe macroscopic emergent phenomena such as the Easterlin paradox and population SWB in terms of behaviour (e.g., rules) of the individual agents (Bonabeau, 2002; Conte and Paolucci, 2014). They often consist of many, typically heterogeneous, agents that interact (spatially, temporally, on social networks) and can adapt to their changing environments (An et al., 2021). Thus, allowing for heterogeneous components and their interactions explicitly. They can systematically link changes at the individual level with their consequences at the aggregate level, how these levels interact with each other, and can analyse the implications of policies on multiple scales (Bruch and Atwell, 2015). This approach allows one to examine how factors such as inequality and reference groups impact SWB at both the individual and the societal level, providing insight into the entangled relationships between income, adaptation mechanisms, social comparison, and SWB. Moreover, there is also evidence that income and SWB are positively associated over time (McGuire et al., 2022; Oparina et al., 2024). This raises questions about under what conditions the paradox is valid. As ABMs are generative models, one can simulate hypothetical conditions or counterfactual scenarios in which the Easterlin paradox can be tested.

Here, we investigate the impact of the Easterlin paradox on individual/societal SWB and resilience using insights from an ABM. Specifically, we explore: a) the short-term dynamics, when individuals experience income growth, they initially feel happier due to improved absolute living standards and positive social comparisons, b) long-term adaptation: over time, individuals adapt to their new income levels (hedonic adaptation), and their social reference group also experiences income growth, leading to a return to baseline SWB levels, and c) relative nature of well-being, influenced by relative income and social position rather than by absolute income levels. The ABM in this study simulates societies where agents are heterogeneous with respect to their SWB set-points and adaptation rates, are subject to income shocks, and interact through social networks and contextual factors (inequality, segregation).

The model yields four key insights. First, social comparison can significantly obstruct full adaptation following economic shocks. Second, the direction of social comparison matters: downward comparisons enhance SWB resilience in the form of full recovery to negative economic shocks, while upward comparisons reduce long-term SWB and increase community-level instability. Third, social comparison mitigates the population-level effects of economic shocks when they impact many individuals but amplifies long-term changes in subjective well-being when only a few are affected, making it a double-edged sword. Fourth, individuals with low income but high SWB set-points are particularly vulnerable to SWB traps and are experiencing significant negative long-term changes and can be trapped in a lower state of SWB after substantial economic shocks. Segregated comparison groups mitigate this effect slightly and can therefore increase resilience. These findings have key implications for targeted and segmented interventions. For example, promoting narratives of shared struggle during widespread crises can leverage downward comparisons. In localised shocks, rapid economic aid should be prioritised to prevent destabilising disparities. In widespread shocks, emphasis should be on communal support systems (e.g., universal basic services) to reinforce solidarity. These insights can aid policymakers to enhance societal resilience while addressing systemic inequities.

## Methods

This section first describes the components of the ABM in detail. To model a population of individuals, we start by describing the dynamics of an individual agent, followed by the network through which the social comparisons take place, and a step-by-step process of a full simulation of the model.

### Individual subjective well-being dynamics

According to adaptation theory (Brickman, 1971), each person has a set-point of SWB around which they fluctuate, which can differ per person and per component affecting SWB (Diener et al., 2009). In this study, we only study one component of SWB and hence only one set-point. As this study focusses on the Easterlin paradox, this component resembles income, which is found to affect SWB (Diener et al., 2018; Easterlin and O’Connor, 2022; VanderWeele, 2017). Undoubtedly, there are more components affecting SWB, and thus this is a very strict assumption. However, we focus on how social conditions (e.g., segregation, inequality) interact with individual characteristics and social structures such as adaptation and social comparison to simultaneously affect the dynamics of SWB. Hence, the modelling of other factors with possibly different set-points is left for future work.

#### Fixed traits and initial values

The variance in SWB between individuals is largely determined by genetic and personality factors (Bartels and Boomsma, 2009; Diener et al., 2018; Pelt et al., 2024). One of the most common findings in SWB research is that extroversion and neuroticism are associated with SWB. Individuals high (low) on the extroversion (neuroticism) scale are found to have higher set-points of SWB and vice versa (Luhmann and Intelisano, 2018). In this study the assumption is that individuals have different SWB set-points. However, the actual relationship between genetics and SWB is not modelled. These differences are drawn from a standard normal distribution (*x*_*i*_ ~ *N*(0, 1)) which are then normalised between 0 and 1. In addition, as set-points are found to be mostly non-neutral (i.e., most individuals are happy) (Diener et al., 2009), we artificially transform the set-points using the following formula: *ln*(*x*_*i*_ + 0.25). Lastly, another normalisation is performed to bound the set-points between 0.05 and 0.95, as perfectly unhappy or happy (i.e., 0 or 1) would be unrealistic. Note that set-points are assumed to be constant over time. This seems plausible, but it should be noted that personality traits such as extraversion and neuroticism (related to genetics) can change (McAdams and Olson, 2010).

**Fig. 1:**
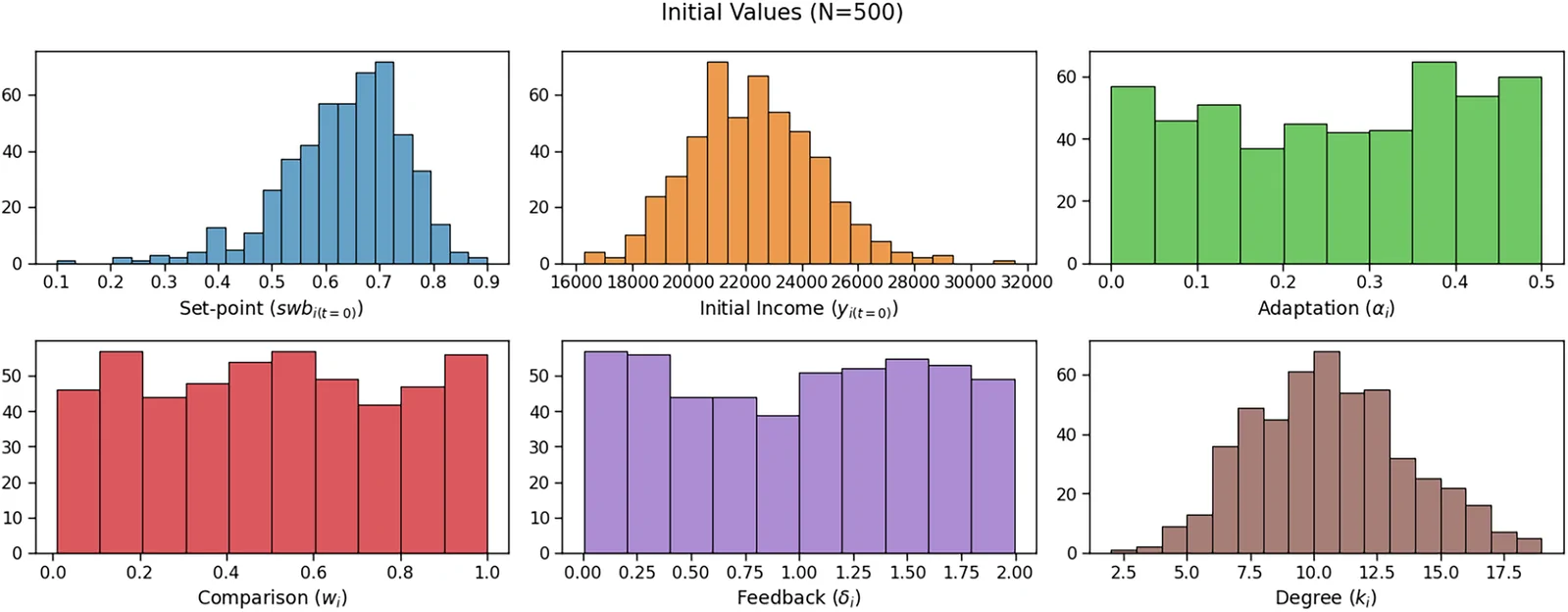
Initial values. Random differences are assumed to influence the SWB set-points of individuals. These differences are drawn from a *N*(0, 1) distribution and normalised between 0 and 1. They are then transformed into non-neutral set-points (*ln*(*x*_*i*_ + 0.25)) and normalised between 0.05 and 0.95. Initial income and the level of inequality is determined by sampling from a *LN*(*μ*, *σ*) distribution (*μ* = 10, *σ* = 0.1 is plotted), adaptation rates are *U*(0, *α*_*max*_ = 0.5) distributed, social comparison weights *w*_*i*_ ~ *U*(0, *w*_*max*_ = 1), feedback strengths *δ*_*i*_ ~ *U*(0, *δ*_*max*_ = 2) and average degree $\widehat{k}=10$.

Initially, individuals start with income levels drawn from a lognormal distribution, *y*_*i*(*t*=0)_ ~ *LN*(*μ*, *σ*), where a larger *σ* would lead to a more unequal society. Adaptation rates, social comparison weights, and feedback strengths are all drawn from uniform distribution with an upper bound that can be varied. These parameters will be described in more detail below, but important is that they can differ between individuals (i.e., heterogeneity). In addition, the degree (*k*_*i*_) is the number of individuals in each comparison group, which can also be varied using the average degree ($\widehat{k}$) of the network (explained in Section “Social comparison network”). Histograms of the different initial distributions can be found in Fig. 1.

#### Adaptation

The hedonic treadmill (Brickman, 1971), also known as hedonic adaptation, is another key component in explaining the Easterlin Paradox. It refers to individual’s tendency to return to a relatively stable level of SWB (their set-point or baseline) despite major positive or negative life changes (i.e., habituation, adaptation). As incomes rise over time, individuals adapt to their new circumstances, and their expectations and desires also increase. This adaptation negates the long-term positive effects of income growth on well-being. Strictly speaking, the hedonic treadmill implies that only temporary changes in SWB are possible, followed by a return to a set-point. Although there is evidence that individuals can fully adapt to certain (substantial) shocks (Anusic et al., 2014), evidence is also present for the contrary (Diener et al., 2009). Long-term and lasting changes in SWB have been found due to unemployment and marriage, for example (Anusic et al., 2014). Thus, set-points can change. This revision, together with four other revisions of the classic hedonic treadmill theory, was proposed in Diener et al. (2009) in light of more recent empirical work. Individuals are also found to have different set-points and adaptation rates compared to others (i.e., heterogeneity), and different set-points for different drivers of SWB. Moreover, these set points are generally non-neutral, the majority of individuals are happy. Finally, there is likely a bidirectional relationship between SWB and income, where they mutually influence each other. Although most studies focus on how income affects well-being (Kahneman and Deaton, 2010; Killingsworth, 2021; Li and Managi, 2023; Wolbring et al., 2013), it is plausible that SWB could also affect income levels over time. For example, happiness and sadness can lead to different economic decision-making (Heerema and Pessiglione, 2025).

**Fig. 2:**
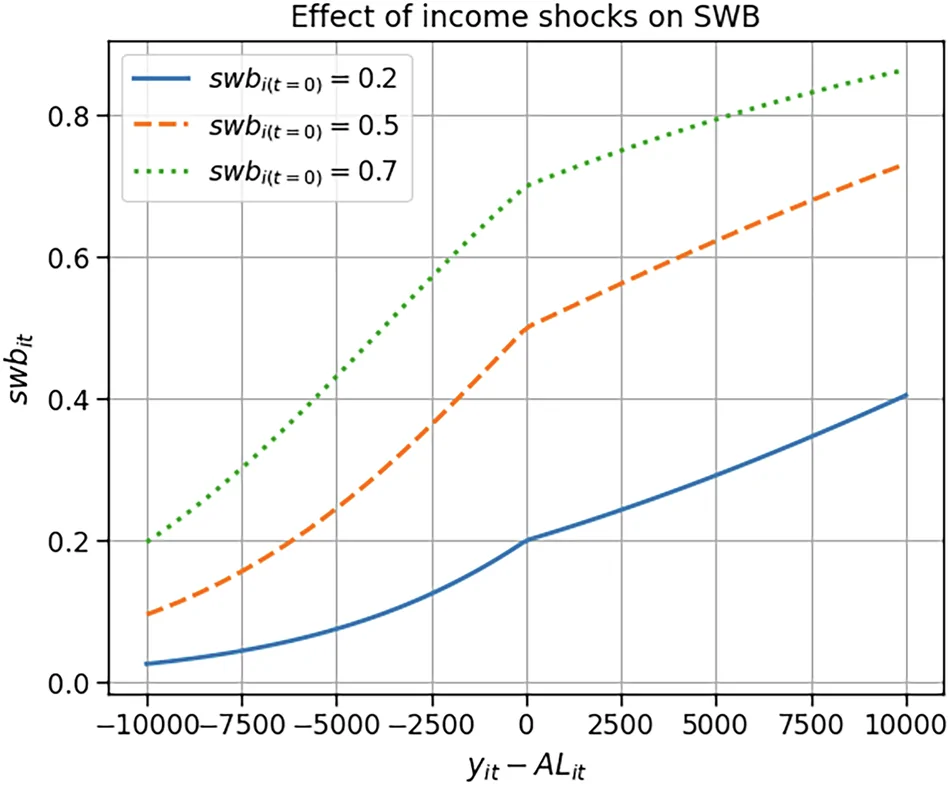
Sigmoid function for different set-points (*swb*_*i*(*t*=0)_). Losses are perceived 2.25 more than gains in accordance with Prospect Theory (Tversky and Kahneman, 1992).

Following adaptation theory (Brickman, 1971), only deviations from your adaptation level can change SWB, for example due to an increase in salary. Suppose a person has an income-level of €100 per month and is fully accustomed to that level, which means that its adaptation level is at €100 (*AL*_*it*_ = 100). If nothing else changes, this person is at its set-point of SWB (*swb*_*i*(*t*=0)_). However, when this person experiences an increase in income, for example, of €20, they experience a (temporary) increase in SWB. However, how strongly an individual reacts in terms of SWB depends on the size of the shock in relation to the adaptation level (*y*_*it*_ − *AL*_*it*_) and its set-point (*swb*_*i*(*t*=0)_). Moreover, according to Prospect Theory (Kai-Ineman et al., 1979), losses are perceived worse than gains and therefore the slope of the sigmoid is different for losses (*λ* = 2.25) than for gains (*λ* = 1). The parameter value of 2.25 is based on a Prospect Theory study (Tversky and Kahneman, 1992). To bound SWB between 0 and 1, a sigmoid function is used that is centred at the individual set-point (Eq. (1), Fig. 2) and a slope of −0.0001 is chosen to have reasonable gains/losses for the income distributions used.

1 $$sw{b}_{it}=\frac{sw{b}_{i(t=0)}}{sw{b}_{i(t=0)}+(1-sw{b}_{i(t=0)}){e}^{{z}_{i}\cdot \lambda \cdot ({y}_{it}-A{L}_{it})}}$$

The exponent controls how strongly the agent reacts to the shock in income. Because individuals with less income might be more sensitive to shocks than those with more income, we recalculate the individual specific slope each time step based on the income of that individual. More specifically, the income gets transformed into a slope *z*_*i*_ ∈ [−0.0001, −0.00001], where the lowest income gets −0.00001 as slope and the highest −0.0001. For the set-point, the assumption is that the set-points are fixed, although there is evidence for the contrary (Diener et al., 2009). Interestingly, due to social comparison, some individuals are actually found to have different stable points of SWB in the simulations, meaning that they can change due to social comparison and hence this need not be modelled separately.

When the current value of *y*_*it*_ exceeds the adaptation level (*AL*_*it*_), SWB gets a positive boost and negative vice versa. However, because of adaptation, the person habituates to this new level over time. The speed of adaptation is dictated by *α*_*i*_, the adaptation rate, and will eventually ensure that the person returns to its set-point level. According to Helson (1948) and Frederick and Loewenstein (1999), the adaptation level is a function of all past stimuli in the following way:

2 $$A{L}_{it}={\alpha }_{i}{y}_{i(t-1)}+(1-{\alpha }_{i})A{L}_{i(t-1)}.$$

Higher rates of adaptation (habituation) mean that a person adapts faster, reflected in larger values of *α*_*i*_ ~ *U*(0, *α*_*max*_). When the adaptation rate equals 1, the agent immediately adapts to the new level in the next time step, experiencing a very short increase in SWB. Note that there are other ways of calculating adaptation levels described in the literature, for example by performing a smoothing function over a certain amount of past stimuli. For example, Schmalz et al. (2007) use a Gaussian filter for this purpose to calculate the adaptation level for a given factor.

#### Feedback

Research also suggests that your SWB can have a feedback effect on the drivers itself. For example, Asebedo et al. (2022) find bidirectional causality between positive emotions and net wealth, after accounting for stable traits. Additionally, in a review Walsh et al. (2023) found that higher initial well-being predicted greater income, job performance, employment likelihood, promotions, and social support. More specifically, adolescents and young adults who report positive affect grow up to earn significantly higher levels of income later in life (De Neve and Oswald, 2012) and hence changes in SWB can be related to increases in income (Bellet et al., 2024). The theoretical foundation for using SWB change comes from Fredrickson’s Broaden-and-Build Theory (Fredrickson, 2004) and “upward spiral” mechanism, one of the most influential frameworks in positive psychology. The theory proposes that positive emotions (experienced momentarily) broaden cognitive and behavioural repertoires, which then build enduring personal resources over time.

**Fig. 3:**
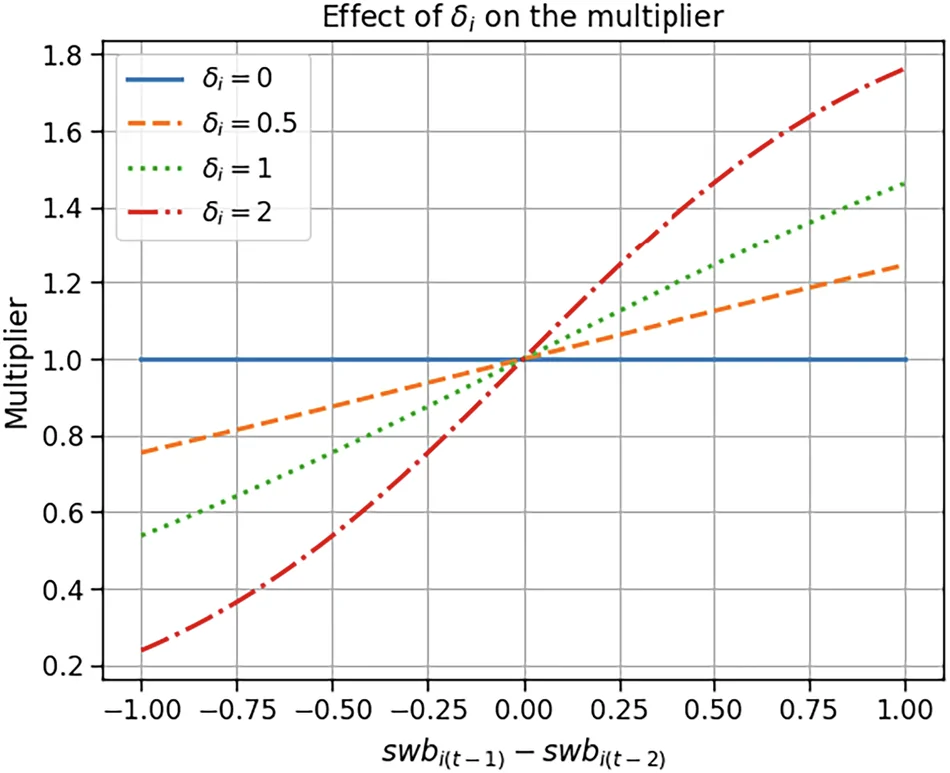
Well-being feedback. Multiplier for different levels of the individual feedback parameter *δ*_*i*_ as a function of the difference in SWB (*swb*_*i*(*t*−1)_ − *swb*_*i*(*t*−2)_).

This is modelled through the multiplicative factor *δ*_*i*_≥0. Whenever an agent experiences a positive boost in SWB (*swb*_*i*(*t*−1)_ − *swb*_*i*(*t*−2)_ > 0), this will reflect itself positively in the income and negatively if *swb*_*i*(*t*−1)_ − *swb*_*i*(*t*−2)_ < 0. These changes are moderated by a sigmoid transformation with decreasing marginal returns (Eq. (3) and Fig. 3). As individuals are likely different in how their current SWB level feeds back into their income, this is drawn from a *U*(0, *δ*_*max*_) distribution. Here *δ*_*i*_ = 2 indicates very strong feedback and *δ*_*i*_ = 0 no feedback at all (Fig. 3).

Moreover, income can grow or decrease over time and is subject to shocks. Random shocks are drawn from a *N*(0, *σ*_*shock*_) distribution and income grows with a growth rate *g*. Multiplication is used to keep the initial level of inequality similar, as the addition of normally distributed shocks would result in normally distributed income (losing the initial distribution shape).

3 $${y}_{it}={y}_{i(t-1)}\cdot \frac{2}{1+{e}^{-{\delta }_{i}(sw{b}_{i(t-1)}-sw{b}_{i(t-2)})}}\cdot (1+g+{\varepsilon }_{it}),{\varepsilon }_{it} \sim N(0,{\sigma }_{shock})$$

**Fig. 4:**
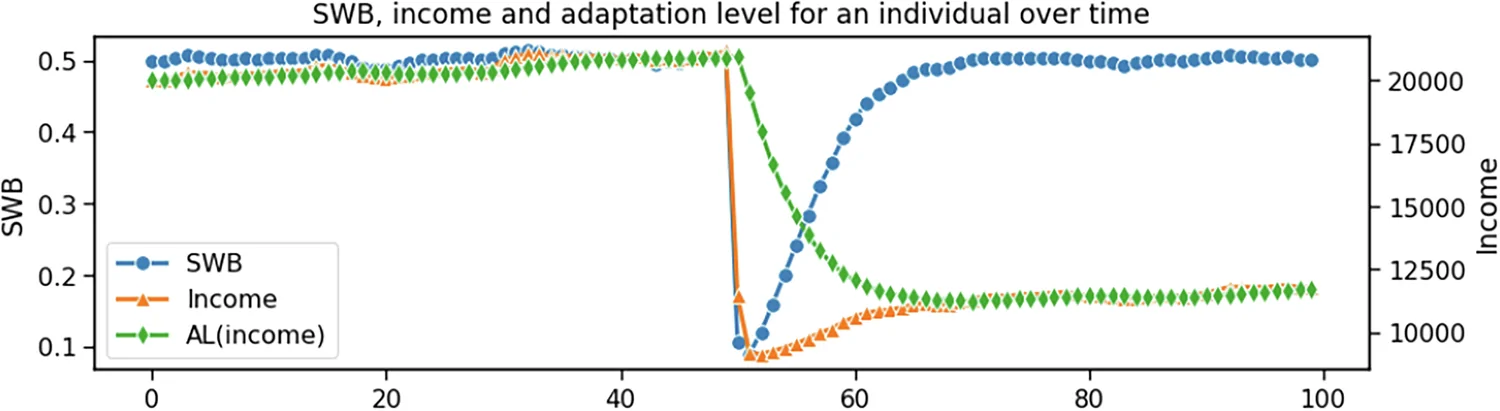
Individual SWB dynamics. An individual with a set-point of 0.5 (*swb*_*i*(*t*=0)_ = 0.5) and an income level of 20,000 experiencing random shocks drawn from *N*(*g* = 0.002, *σ*_*shock*_ = 0.005) and a shock of −10,000 at *t* = 50. The negative shock leads to a decrease in income, resulting in a decrease in SWB. Over time the individual habituates to the new level of income (*α*_*i*_ = 0.15, *δ*_*i*_ = 1) and SWB returns to its set-point.

Figure 4 shows a realisation of individual dynamics of SWB. In addition to experiencing random shocks with a *σ*_*shock*_ = 0.001 in every time step and income growth (*g* = 0.002), the agent receives a large negative shock of size 10,000 at *t* = 50. Due to this shock, income decreases significantly. Because the individual does not adapt immediately, SWB is also affected, but, over time, the individual adapts to the new (lower) level of income and hence returns to its set-point of SWB.

### Societal subjective well-being dynamics

Individual SWB dynamics do not take place in isolation, there is ample evidence of social structures influencing SWB and vice versa (Brown et al., 2008; Easterlin, 1974; Gerber et al., 2018; Helliwell and Aknin, 2018). An explanation of the Easterlin paradox is social comparison (Easterlin, 1974; Easterlin and O’Connor, 2022; Wolbring et al., 2013). At a point in time, those with higher income are happier because they are comparing their income to that of others who are less fortunate, and conversely for those with lower income. However, over time, as income increases within the population, the income of the comparison group also increases, negating the otherwise positive effect of individual income growth on SWB (Easterlin and O’Connor, 2022). Among others, Smith et al. (1989), Hagerty (2000) and Ferrer-i Carbonell (2005) find that social comparison is the main cause of the paradox. Taking this one step further, if social networks are stratified and therefore segregated (McPherson et al., 2001), it is likely that individuals will interact with others like them. As these similarities are often related to income, one is likely to compare with individuals with similar income levels. In addition, it is not only important that individuals compare themselves to others, it also matters how they do it. In a meta-analysis of social comparisons, Gerber et al. (2018) find that individuals tend to mainly engage in upward comparisons, with self-deflating effects as a result.

#### Social comparison network

To generate social networks that can vary both in their level of segregation and the number of individuals to whom one compares (degree, *k*_*i*_), the social distance attachment (SDA) model is used (Boguná et al., 2004). The SDA model uses the (social) distance between agents to calculate connection probabilities. More specifically, each pair of nodes has a probability of connection *p*_*ij*_ which is given by:

4 $${p}_{ijt}={\left(1+\frac{{[d({y}_{i(t=0)},{y}_{j(t=0)})]}^{h}}{{\beta }_{SDA}}\right)}^{-1},$$

In which *β*_*SDA*_ is the characteristic distance, *h* is the level of homophily and *d*( ⋅ ) is the Euclidean distance between nodes *i* and *j* (based on their income). The higher the value of the homophily parameter, the more segregated the network will be. Hence, segregation is explicitly modelled as an outcome of choice homophily, the tendency to connect to similar others, and differences between individuals (i.e. income inequality). However, segregation can arise in various other ways, which we will not dive into here.

Although the SDA model is very closely related to Fermi-Dirac preferential attachment, SDA is preferred for this case, as any desired amount of expected connections ${\mathbb{E}}[k]$ can be computed by setting *β*_*SDA*_ and the amount of expected connections can be easily computed by adding the connection probabilities and dividing by the number of nodes:

5 $${\mathbb{E}}[k]=\frac{1}{N}\mathop{\sum }\limits_{i=1}^{N}\mathop{\sum }\limits_{j=1}^{N}{p}_{ij}$$

Another advantage of SDA is that distribution of the distances between the nodes does not have an influence on the level of homophily. This implies that only the homophily parameter *h* dictates the level of segregation in the network (Talaga and Nowak, 2019). When *h* and the average degree $\widehat{k}$ are chosen, *β*_*SDA*_ is optimised from their respective values (Boguná et al., 2004). Resulting levels of network assortitivity (i.e., a measure of network segregation) for different values of homophily and the average degree can be found in Supplementary Fig. A1.

#### Mechanisms of social comparison

Social comparison is the process of evaluating one’s own situation relative to others. When it comes to SWB, income relative to others is found to be more important than absolute income levels (Easterlin and O’Connor, 2022; Lakshmanasamy and Maya, 2020; Paul and Guilbert, 2013; Wolbring et al., 2013). As incomes rise throughout the population and the incomes of one’s comparison group also increase, it negates the positive effect of individual income growth on SWB. Moreover, we tend to make upward comparisons with those who “have more” or who “do better”, which can negatively affect SWB (Gerber et al., 2018). There is also evidence that it is not relative income that matters for SWB, but the rank in a comparison group does (Boyce et al., 2010; Brown et al., 2008; Hopkins and Kornienko, 2009; Osafo Hounkpatin et al., 2015). The above evidence indicates that increase in subjective well-being (SWB) occurs only when a rise in income translates into a higher relative rank. Moreover, this rank-based comparison is found to be stronger in more unequal countries (Macchia et al., 2020), implying an interaction with inequality. Conceptually, social income, which encompasses social networks and relationships, provides the context for these relative comparisons. As individuals’ social connections expand, they may compare themselves to a wider range of individuals, potentially affecting their SWB. Although studies have started to incorporate social comparison into dynamic models of SWB (Baggio and Papyrakis, 2014), key social structures affected by homophily and segregation^2^ are often neglected (Gerber et al., 2018; McPherson et al., 2001). In segregated environments one’s reference group is likely to be more similar to the comparer than in less segregated contexts. This can affect the resilience and robustness of individuals and communities when the comparer receives a large shock while its reference group does not.

Social comparison effects are modelled through the adaptation level of an agent (*AL*_*it*_). This depends not only on its own past income levels as in Eq. (2), but also on its reference group. In this study, we assume that an individual compares itself to a specific quantile in its reference or comparison group (Eq. (6)) that can be varied but is the same for all agents. For example, when *q* = 0 it means a comparison with the minimum in its reference group, *q* = 0.5 the median, and *q* = 1 the maximum income. The social comparison mechanism works as follows. First, it is assumed that the individual knows all the current income values of its reference group and is able to sort them (Eq. (7)). From this set, the value is chosen that is closest to the chosen quantile *q* (Eq. (8)) to compare. Hence, the individual *i* now has its own previous income value (*y*_*i*(*t*−1)_) and the comparison value of its reference group (*Q*_*i*_(*q*)). The social comparison weight (*w*_*i*_), drawn from a *U*(0, *w*_*max*_) distribution, of Eq. (6) then determines the weight an individual places on social comparison relative to adaptation/habituation to their own (past) income (*y*_*i*(*t*−1)_). Combined with the previous adaptation level (*AL*_*i*(*t*−1)_), this determines how the adaptation level will change over time. When an individual places more emphasis on social comparison, it has a large weight, and thus its level of adaptation strongly depends on the income of its connections. It should be noted that, for example, when *q* = 0.7, the individuals below this quantile in terms of income are making an upward comparison, while those above are comparing downward. This social comparison mechanism is validated with empirical data in Supplementary Figs. E1, E2.

6 $$A{L}_{it}={\alpha }_{i}[(1-{w}_{i})\cdot {y}_{i(t-1)}+{w}_{i}\cdot {Q}_{it}(q)]+(1-{\alpha }_{i})A{L}_{i(t-1)}$$

7 $${Y}_{i}=\{{y}_{jt}| {a}_{ijt}=1\},\,{\rm{such\; that}}\,{y}_{1t}\le {y}_{2t}\le ...\le {y}_{{k}_{i}t}$$

8 $${Q}_{it}(q)={Y}_{i}^{(m)}\,\,{\rm{where}}\,\,m=\lfloor q\cdot {k}_{i}\rceil$$

### Simulation

**Fig. 5:**
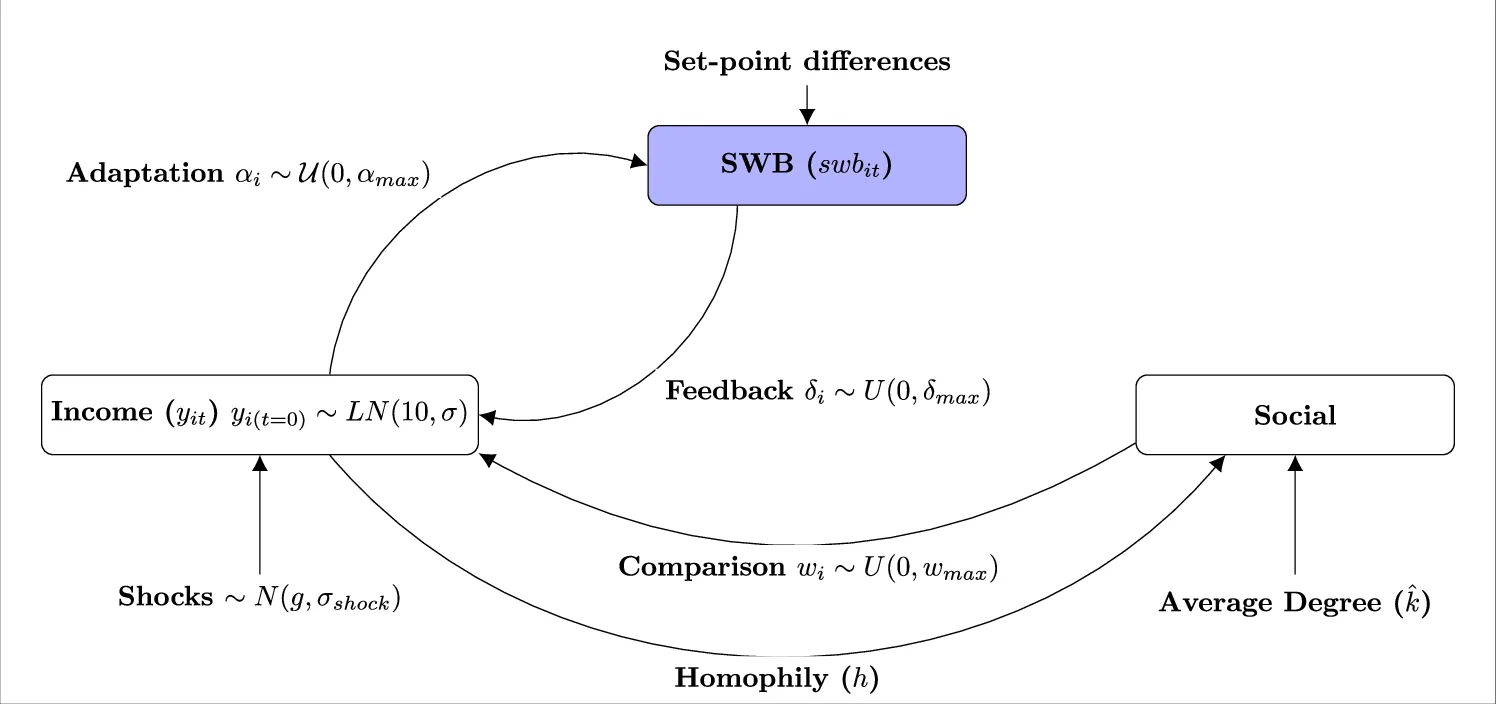
Model diagram. The diagram indicates how the factors affect each other and what parameters are directly influencing the factors or effects. Parameters with no subscript are fixed throughout the simulation and the same for every agent, while subscript *i* indicates its different for every individual and *it* if it also varies over time.

The society is simulated for 100 time steps with a relatively large shock of size −0.25 at *t* = 50 to the income of a fraction (*p*) of the agents. The magnitude of the perturbation is mainly to exacerbate the effects, but in reality (perceived) shocks can vary between individuals. In addition, at each time step, the agents receive a smaller random shock sampled from a *N*(0, *σ*_*shock*_) distribution. The adaptation rates are randomly drawn from a uniform distribution with bounds of 0.05 and 0.15, while initial income is assumed to form a Beta distribution (*B*(*a*, 10) + 1), where *a* can be varied to model inequality. Algorithm 1 gives a step-by-step guide of a simulation (i.e., model run) and Fig. 5 presents the most important components of the model. See Supplementary Fig. A4 for a simple example society with only three agents.

#### Algorithm 1

Simulation of subjective well-being dynamics

1: **Initialisation:** Set SWB set-points, income, feedback, social comparison weights, and adaptation rates (see Section “Fixed traits and initial values”).

2: Create the network using distances between agents’ initial income, average degree $\widehat{k}$, and homophily *h*.

3: Copy network and initial values from *t* = 0 to *t* = 1 (as Eq. (3) depends on two previous steps).

4: Apply stochastic shocks: ${\mathcal{N}}(0,{\sigma }_{shock})$ for all agents at each time step. At *t* = 50, apply a deterministic negative shock of −10,000 to a fraction *p* of the population.

5: **for** *t* = 3 **to** 100 **do**

6: Update income using Eq. (3)

7: Update adaptation levels with social comparison (Eq. 2)

8: Update SWB using Eq. (1)

9: **end** **for**

### Output metrics

The system is analysed on two levels, the individual and the population, as well as using three different measures. All metrics are first calculated on the individual level before taking population averages. Firstly, long-term changes in SWB are calculated to see if the large negative perturbation has resulted in any long-lasting changes in SWB. Since agents are also randomly shocked in every time step, for each agent an average is taken over the SWB of the 20 time steps before the large shock (at *t* = 50) and for the last 20 (*t* = 80 until *t* = 99). Subtracting the former from the latter gives an indication of how well the agents have adapted to the large shock. Negative values indicate that agents did not fully adapt and have (long-lasting) lower levels of SWB.

9 $$\mathrm{Long}-{\mathrm{term\; change}}_{i}=\frac{1}{20}\mathop{\sum }\limits_{t=80}^{99}sw{b}_{i}-\frac{1}{20}\mathop{\sum }\limits_{t=30}^{49}sw{b}_{i}$$

Furthermore, to assess the (in)stability of an individual, the coefficient of variation (CV) is calculated. The standard deviation of SWB of an individual is divided by the mean of SWB over the course of an entire simulation (i.e., 100 time steps). If the CV is low, the individual is relatively stable. Note that agents who are shocked will have a larger CV than those who are not if all else is equal.

10 $${\mathrm{Instability}}_{i}=\frac{\sqrt{{\sum }_{t=0}^{99}{(sw{b}_{i}-\frac{1}{100}\mathop{\sum }\limits_{t=0}^{99}sw{b}_{i})}^{2}}}{\frac{1}{100}\mathop{\sum }\limits_{t=0}^{99}sw{b}_{i}}$$

Toward a more dynamic measure of SWB, we also calculate the time it takes for an individual to return to its previous temporally stable state just before the shock at *t* = 50. We define stability as the first time step (after *t* = 50) when an individual is within 5% of its level of well-being at *t* = 49 (*swb*_*i*(*t*=49)_).

11 $${\mathrm{Recovery\; time}}_{i}=\min \{{t}_{i}^{* }\ge 50:\frac{| sw{b}_{i(t={t}^{* })}-sw{b}_{i(t=49)}| }{sw{b}_{i(t=49)}} < 0.05\}-50$$

The three output measures can be thought of as aspects of resilience. For example, Schäfer et al. (2024) define resilience as “the maintenance or fast recovery of mental health during or after stressor exposure”. Maintenance can be captured with long-term changes or a low CV, while fast recovery would be reflected in the recovery time. Similarly, Galatzer-Levy et al. (2018) describe it as “stable psychological health before and after the traumatic event”. Where stability is captured by the CV.

## Results

Although the model’s components draw credibility from their foundation in empirical and theoretical literature, we enhance its validity by demonstrating its ability to replicate several key empirical phenomena in the next section and a validation of the micro-rules in the Appendix. Building on this, we investigate how social comparison shapes long-term shifts in subjective well-being, individual instability, and recovery. Finally, we explore how varying initial conditions, which represent diverse social contexts, systematically impact these outcomes.

**Fig. 6:**
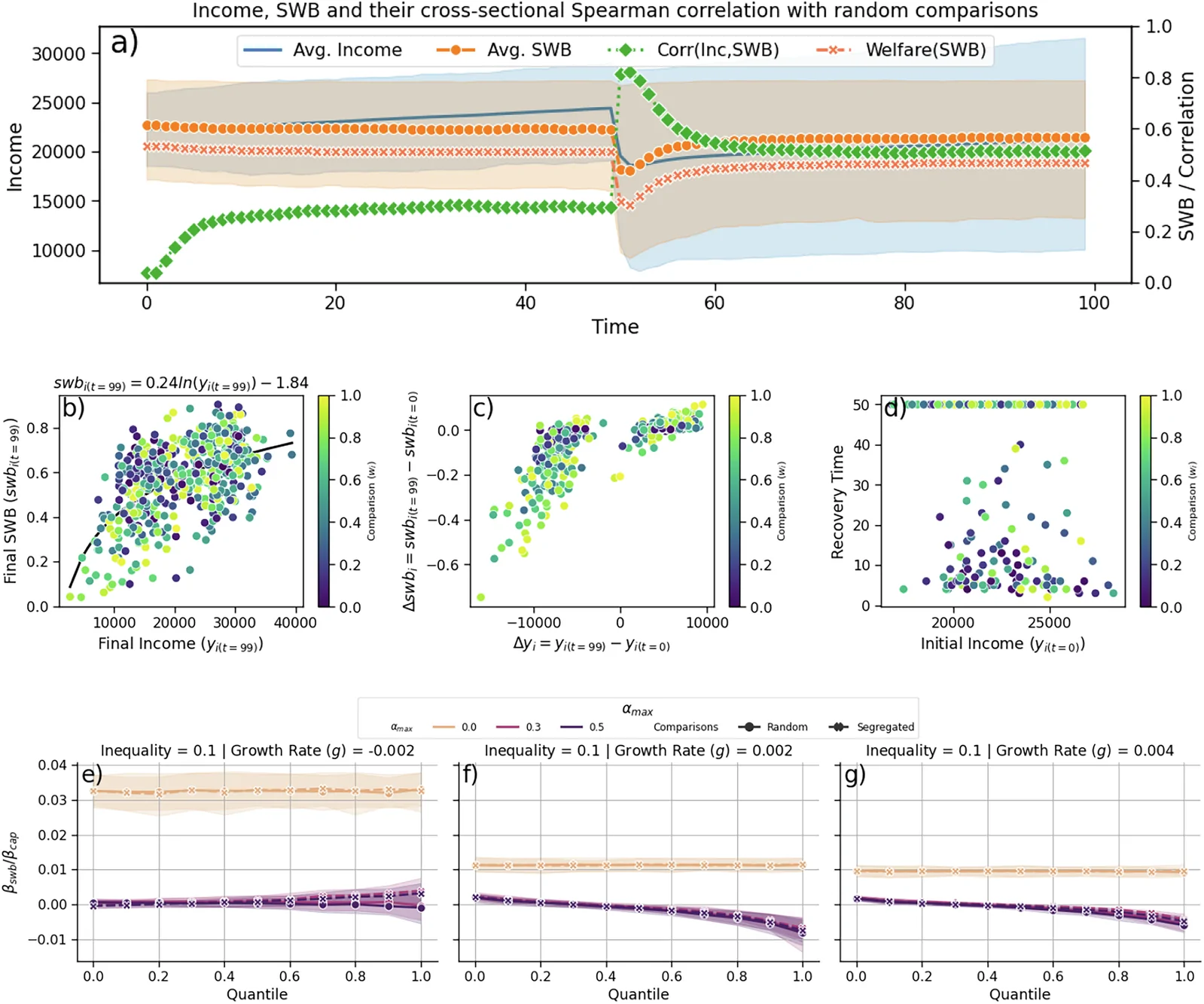
Stylised facts. **a** Well-being, income and their Spearman correlation at every time step with random comparison groups ($\widehat{k}=10$). Bounds indicate the 5% and 95% percentile and 50% of the individuals receive a negative shock at *t* = 50. Other fixed parameter values can be found in Table 1. **b** Income and SWB in the last time step. **c** The change in SWB over the course of the full simulation as a function of the change in income. **d** The recovery time as a function of initial income for people that are shocked. **e**–**g** Show the ratio of two regression coefficients, where average SWB is predicted by *t*: $\overline{sw{b}_{it}}={c}_{swb}+{\beta }_{swb}\cdot t$) *β*_*swb*_ and average income by: $\overline{{y}_{it}}={c}_{cap}+\frac{{\beta }_{cap}}{1000}\cdot t$). These regressions are based on observations from *t* = 50 onwards, to avoid initial changes affecting the estimation. Simulations are with *p* = 0.5 for (**a**–**d**) and no shock (*p* = 0), *σ* = 0.8 for (**e**–**g**). Other fixed parameter values can be found in Table 1.

In Fig. 6a, one can see that after an initialisation phase, income is positively correlated with SWB, as indicated by the dotted line^3^. However, over time, average income and inequality are increasing, but this does not result in any increase in average SWB. Hence, income and SWB are not positively associated over time. This can be seen more clearly in Fig. 6b, c). Where in b), those with more income are likely to have higher SWB, but c) shows that the change in income over the course of the full simulation does not lead to a corresponding change in SWB for most agents (only those who are shocked end up with lower SWB). These two facts together form the Easterlin paradox as explained previously. Another outcome of the model is that initial income affects the recovery time of individuals (Fig. 6d). Having a lower starting income results in longer recovery times, this is in alignment with the findings of a systematic review of Schäfer et al. (2024), which identified individual income as the main determinant of resilience^4^ at the individual level.

Part of the Easterlin Paradox can be summarised by the ratio of two regression coefficients ($\frac{{\beta }_{swb}}{{\beta }_{cap}}$), the numerator comes from the prediction of average SWB: $\overline{sw{b}_{it}}={c}_{swb}+{\beta }_{swb}\cdot t$ and the denominator from predicting average income by $\overline{{y}_{it}}={c}_{cap}+\frac{{\beta }_{cap}}{1000}\cdot t$). The ratio should be zero for the paradox to hold and if it does not, it gives the degree of the violation. Figure 6e–g shows the ratio for different quantiles, growth rates, and adaptation levels (i.e., *α*_*max*_). In case of no adaptation and economic decline (*g* < 0) the ratio of slopes is positive as both slopes are negative and when *g* > 0 both average income and average SWB increase over time, violating the paradox. When individuals do adapt to shocks over time (*α*_*max*_ > 0), the ratio is very close to zero, and hence the paradox holds. However, increasing the quantile has negative effects, it decreases average SWB over time (the numerator is negative in all cases) for both negative and positive growth rates. Hence, adaptation with respect to economic growth is a necessary ingredient for the paradox to hold, as already indicated by Easterlin and O’Connor (2022) among others, whereas the social comparison is necessary for positive correlation in cross-sectional data. In addition, comparing to the higher quantiles seems to result in a violation of the paradox in this model. This effect reduces the faster one adapts in case of negative growth rates but is more severe for economic growth.

### Long-term effects of social comparisons on well-being

**Fig. 7:**
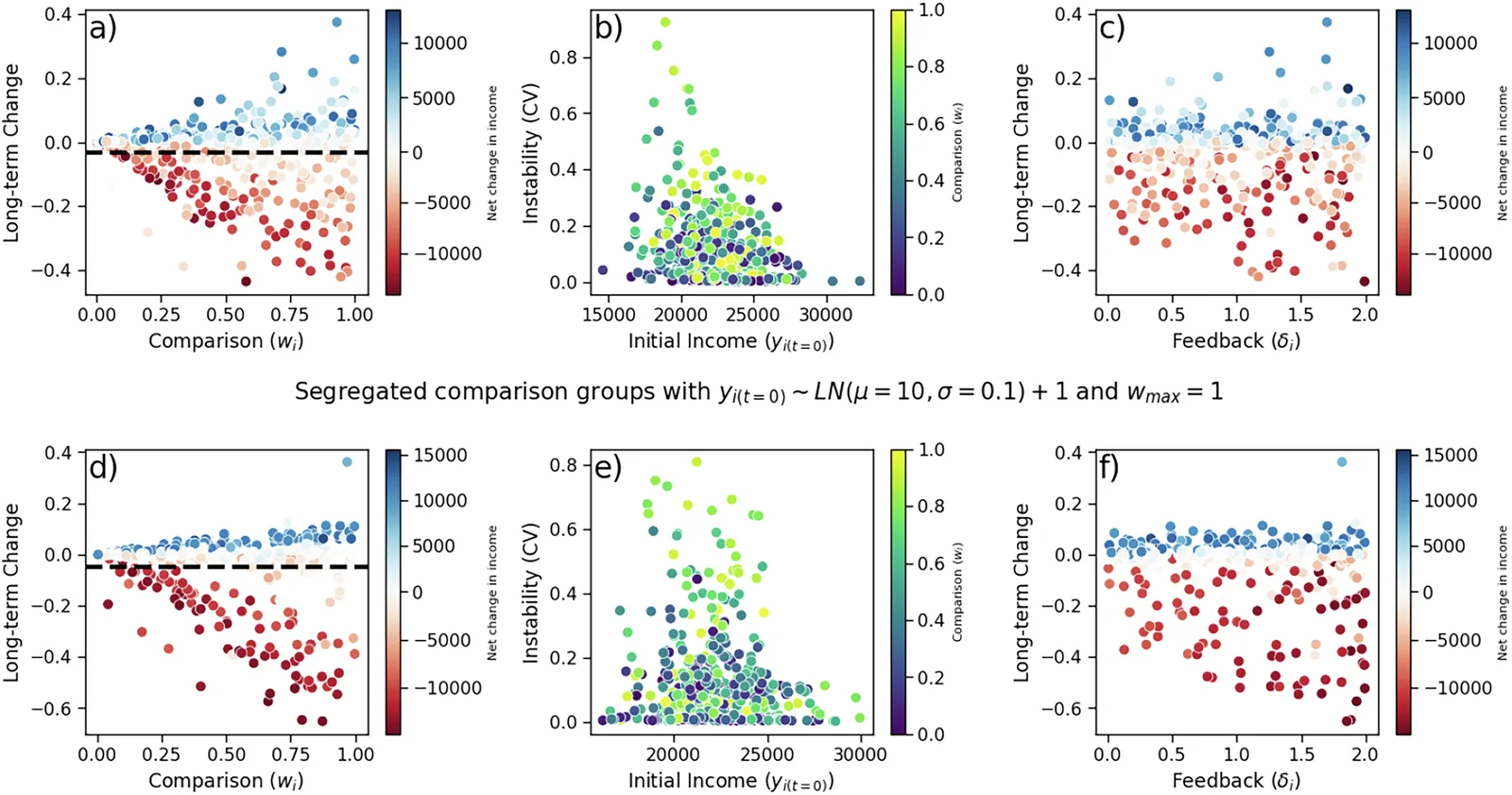
Social comparison. A simulated society (*N* = 500) with initial income distributed as *LN*(*μ* = 10, *σ* = 0.1) where individuals have on average 10 random (**a**–**c**) or segregated (**d**–**f**) connections and compare themselves to the 50% percentile (*q* = 0.5). The long-term changes in SWB as a function of the social comparison weight (**a**, **d**) and the hue, “net change'', is defined as: (*y*_*i*(*t*=51)_ − *Q*_*i*(*t*=51)_) − (*y*_*i*(*t*=49)_ − *Q*_*i*(*t*=49)_). The instability is measured by the coefficient of variation and is plotted as a function of the feedback parameter (**b**, **e**). The recovery times are shown in relation to the social comparison weights (**c**, **f**). All agents receive small perturbations ~ *N*(*g* = 0.002, *σ*_*shock*_ = 0.005) at every time step and 50% gets a shock of −10,000 at *t* = 50. Other fixed parameter values can be found in Table 1.

Here we demonstrate the impact of social comparison on SWB. Figure 7a, d reveals that social comparison mechanisms can drive significant long-term changes in SWB. Although individual agents are designed to fully adapt to shocks, as dictated by the adaptation theory (Fig. 4), the influence of social comparisons disrupts this process. Notably, a higher social comparison weight (*w*_*i*_) amplifies these long-term effects. For instance, among a population where 50% experience negative shocks, the majority suffer a decline in SWB. However, those spared from shocks often see an increase in SWB, driven by shifts in their comparison groups. This dynamic aligns with emerging empirical studies, such as Diener et al. (2009), which revise classical adaptation theory to recognise conditions under which SWB does not fully stabilise.

Despite these findings, not all individuals exhibit long-term SWB changes, even when *w*_*i*_ is high. This raises a pivotal question: what factors determine the extent of SWB changes in agents? To better understand this phenomenon, the hue in Fig. 7a, d, labelled as “net change”, is defined as:

$$\,{\rm{Net\; change}}\,=({y}_{i(t=51)}-{Q}_{i(t=51)})-({y}_{i(t=49)}-{Q}_{i(t=49)}).$$

This metric captures the shift in an individual’s income from *t* = 49 to *t* = 51, relative to the income of their comparison group. The two-step interval accounts for the delayed effect of changes in others’ income on an individual’s adaptation level. If the net change is negative, it strongly indicates a persistent negative impact on SWB.

For example, consider an individual with less income who compares themselves to the maximum in their group (*q* = 1) and experiences a negative shock. At *t* = 51, their income (*y*_*i*(*t*=51)_) is lower than at *t* = 49, deepening the gap between current income and their adaptation level, as defined by Eq. (1). This disparity is exacerbated for individuals with a high *w*_*i*_, intensifying long-term SWB shifts. Conversely, if shocks also affect others within the comparison group, the redistribution of comparison benchmarks can mitigate these effects. This dynamic resonates with empirical evidence supporting the “social normativity” hypothesis. For instance, in the context of unemployment, its psychological impact is less severe when widespread, as reduced stigma, shame, and guilt alleviate its negative effects (Torche and Daviss, 2025). In summary, the interplay of social comparison and individual adaptation underscores the complex pathways through which SWB evolves, challenging conventional theories of complete adaptation and opening avenues for nuanced exploration of social dynamics.

In social networks with random comparison groups (*h* = 0), individuals with a lower initial income experience greater instability in SWB (Fig. 7b). However, this effect diminishes in segregated networks, as shown in Fig. 7e. The increased instability in random networks stems partly from larger disparities in income within comparison groups. These disparities drive more substantial shifts in individuals’ adaptation levels during the initial steps of the simulation, triggering pronounced changes in SWB (Supplementary Fig. C1a).

This finding highlights the role of broader societal conditions in shaping the dynamics of the model, a theme explored further in Section “Impact of segregation and inequality on well-being”. Additionally, individuals with stronger feedback effects (i.e., higher *δ*_*i*_) exhibit more significant long-term changes in SWB, underscoring the interaction between individual sensitivity and social context in driving well-being outcomes.

The quantile comparison mechanism leads agents with less income than *Q*_*it*_(*q*) to compare upward and those with more to compare downward. Hence, to study how these groups differ in their SWB, we separate those who compare upward 90% or more of the time steps, those who compare downward for 90% or more, and all others. In addition, to assess the effect of income, individuals are divided into two groups: 50% with the lowest income and 50% with the highest, recalculated every step. Note that for these figures *σ* = 0.8 to enlarge the (initial) differences between the groups.

Significant changes in SWB emerge during the initial steps of the simulations (Supplementary Fig. C1), driven by the adjustment of adaptation levels to newly assigned comparison groups. At the outset (*t* = 0, *t* = 1), initial values are independently set, leading to notable shifts. Predictably, individuals with high income who predominantly engage in downward comparisons benefit the most during these early stages. Conversely, those with low income, primarily engaged in upward comparisons, face initial disadvantages. These effects are amplified by the chosen level of inequality (*σ* = 0.8) and the random network structure, which introduces greater income variability both across the system and within comparison groups.

In a random network, low-income individuals with upward comparisons are likely to benchmark against wealthier counterparts, while high-income downward comparers gain relative advantages. By design, random networks exhibit larger income disparities in comparison groups compared to segregated networks. Over time, as income grows at an average rate of *g* per time step, these disparities expand more rapidly in random networks than in segregated ones.

It is important to note that long-term changes in SWB, calculated using Eq. (9), rely on the average of time steps 30–49 and thus do not fully capture these early shifts. Nonetheless, some long-term patterns are evident. In the random network (Supplementary Fig. C1a), upward comparisons lead to sustained negative changes in SWB, particularly for low-income individuals, whereas high-income groups are less affected. For downward comparers, both low- and high-income groups eventually adapt to the shock, although low-income individuals recover more slowly.

In contrast, segregated networks (Supplementary Fig. C1b) exhibit different dynamics. Neither low- nor high-income groups achieve full adaptation to shocks under upward or mixed comparisons. Additionally, shocks in segregated networks produce larger long-term changes in SWB (Eq. (9)) compared to random networks. This effect arises because comparison groups in segregated networks are more homogeneous in income distribution. When a shock occurs at *t* = 50, it significantly alters the comparison quantile within these more uniform groups, amplifying its impact. These findings illustrate how network structure and initial inequalities shape both short- and long-term SWB trajectories.

### Impact of segregation and inequality on well-being

Previous analyses only considered simulations with one specific initial distribution of income and comparisons on a random or segregated network. To analyse how long-term changes, instability, and recovery times are affected by different initial (or societal) conditions, sensitivity analyses are performed. However, in order to have a more focused sensitivity analysis, a global sensitivity analysis (GSA) is conducted first. In a GSA, all parameters are varied simultaneously to assess which subset of parameters is most important with respect to the output metrics. Supplementary Figs. B1, B2, B3, B4 show that the quantile, growth rate, and upper limits of the adaptation rates and social comparison weights are the most influential parameters according to the PAWN (Pianosi and Wagener, 2015) sensitivity indices for the average long-term change in SWB. Segregation and feedback are important to a lesser extent. For the instability of SWB, as measured by the average of the individual coefficients of variation, it is the same four parameters plus the upper bound of the feedback parameter (*δ*_*max*_) and the inequality (*σ*). For the average recovery times, it is again the first four parameters mentioned, plus the inequality parameter. Hence, in the following sections the focus lies on these parameters with random or segregated comparison groups, with the rest of the parameters having the fixed values described in Table 1. Although an influential parameter, the growth rate is kept fixed at 0.002. This is because the main reason for its influence is when individuals do not adapt quickly enough (low *α*_*max*_) relative to income growth (*g*), and most individuals are converging to a SWB of 1.

**Table 1:** Parameters.

Parameter	Fixed value	Values in GSA
Nr. of agents (*N*)	500	500
Social comparison weight (*w*_*i*_)	*U*(0, *w*_*max*_)	Follows from below
Upper bound for *w*_*i*_(*w*_*max*_)	1	*U*(0, 1)
Adaption rate (*α*_*i*_)	*U*(0, *α*_*max*_)	Follows from below
Upper bound for *α*_*i*_(*α*_*max*_)	0.5	*U*(0, 0.5)
Set-points of SWB (*swb*_*i*(*t*=0)_)	–	–
Feedback of swb into income (*δ*_*i*_)	*U*(0, *δ*_*max*_)	Follows from below
Upper bound for *δ*_*i*_(*δ*_*max*_)	2	*U*(0, 3)
Initial income (*y*_*i*(*t*=0)_ ~ *LN*(*μ* = 10, *σ*))	10	10
Level of inequality (*σ*)	0.1	*U*(0, 0.01)
Income shocks ~ *N*(*g*, *σ*_*shock*_)	0.002	*U*(0, 0.01)
Standard deviation of shocks (*σ*_*shock*_)	0.005	*U*(0, 0.01)
Homophily (*h*)	Random (*h* = 0)	*U*(0.2, 2)
Quantile to compare to (*q*)	0.5	*U*(0, 1)
Average degree ($\widehat{k}$) in the network	10	*U*(2, 10)
Fraction of agents shocked at *t* = 50	0.5	0.5

Fixed values of the parameters and their respective ranges in the GSA. If not stated explicitly otherwise, plots are based on these fixed values of the parameters. The lower bound of the homophily parameter is set to 0.2 as for lower values the algorithm does not converge. In simulations where *h* = 0 is used, an random network is constructed using a different algorithm but with the same average degree as the SDA model.

#### Social comparison, segregation and inequality

**Fig. 8:**
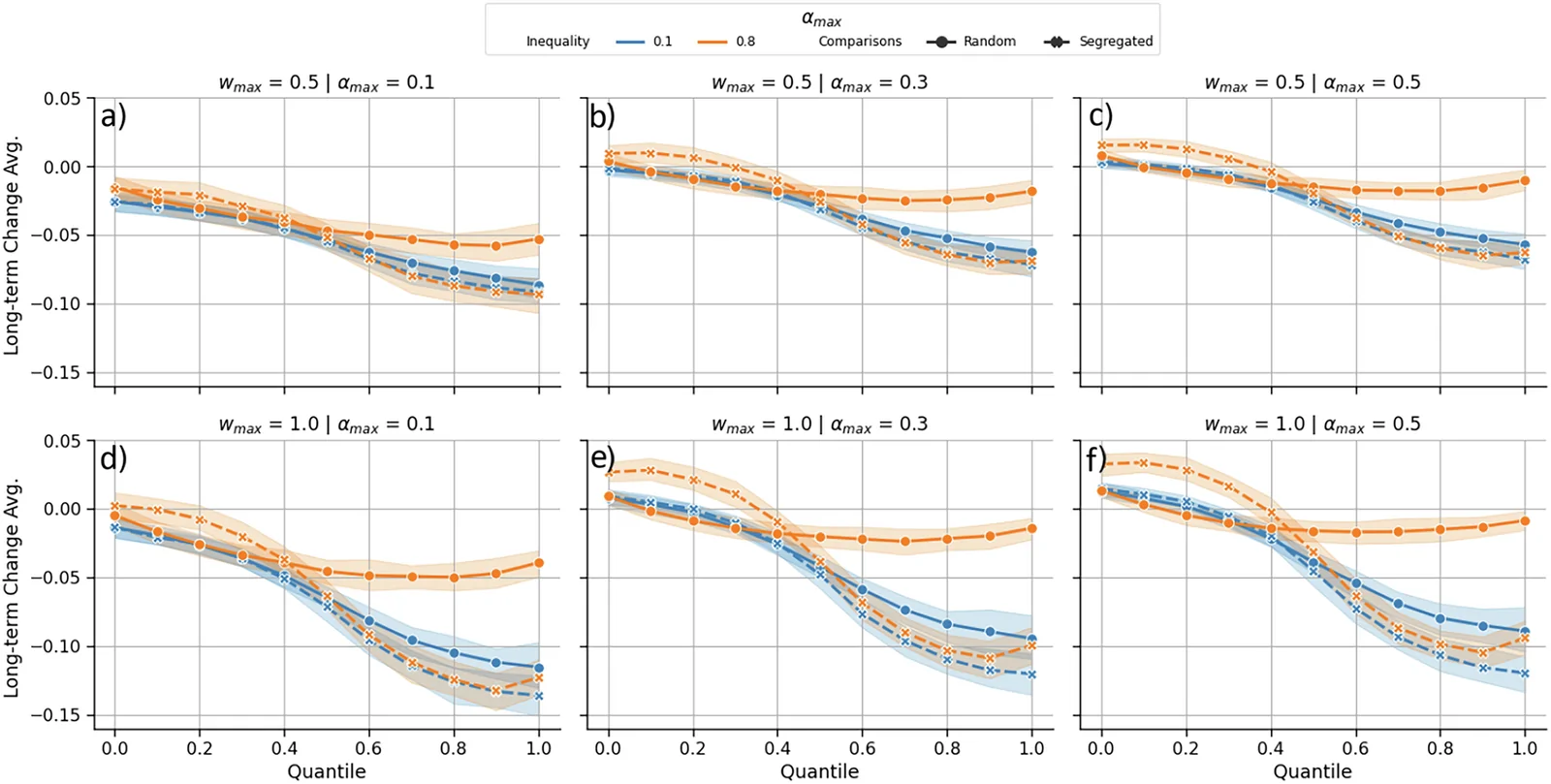
Sensitivity analysis. **a** Average long-term change as a function of the quantile for a maximum social comparison weight of 0.5 and the lowest maximum adaptation rate. **b** Same as (**a**), with an intermediate maximum adaptation rate. **c** Same as (**a**), with the highest maximum adaptation rate. **d** Average long-term change as a function of the quantile for a maximum social comparison weight of 1 and the lowest maximum adaptation rate. **e** Same as (**d**), with an intermediate maximum adaptation rate. **f** Same as (**d**), with the highest maximum adaptation rate. Average long-term changes as a function of the quantile one compares to, two different comparison groups (e.g., random, segregated), the level of inequality (*σ*) and the upper bounds of the adaptation rate (*α*_*max*_) and social comparison weights (*w*_*max*_). The plots are based on 500 agents and 100 replications per parameter configuration and the bounds signify the 5% and 95% percentile. Fixed parameter values can be found in Table 1.

As indicated by the GSA, the quantile and the upper bound of the social comparison weight are two of the most important parameters for the long-term change in SWB. Figure 8 shows that the higher quantile used in the comparison mechanism and the greater emphasis on social comparison (*w*_*max*_ = 1), the more negative the average SWB in the population. Moreover, when individuals adapt at a slower rate (i.e, lower *α*_*max*_), the average long-term change is also more pronounced.

Interestingly, in a relatively unequal society (*σ* = 0.8) with random comparison groups, there is a minimum around *q* ∈ {0.7, 0.8} and the average long-term changes for *q* > 0.5 are substantially less severe than for the other simulated conditions. In addition, the standard deviations of the long-term changes (Supplementary Fig. C3) are not larger than in the other scenarios. Combining these two findings, it means that in the random-unequal case there are substantially lower long-term changes for both individuals and the population as a whole. In contrast, the instability increases substantially in the random-unequal scenario, especially for large *q* (Supplementary Fig. C4). Moreover, the average recovery times are mainly governed by the quantile, showing a linear increase with increasing *q* (Supplementary Fig. C5).

#### Influence of shock prevalence on long-term changes in well-being

**Fig. 9:**
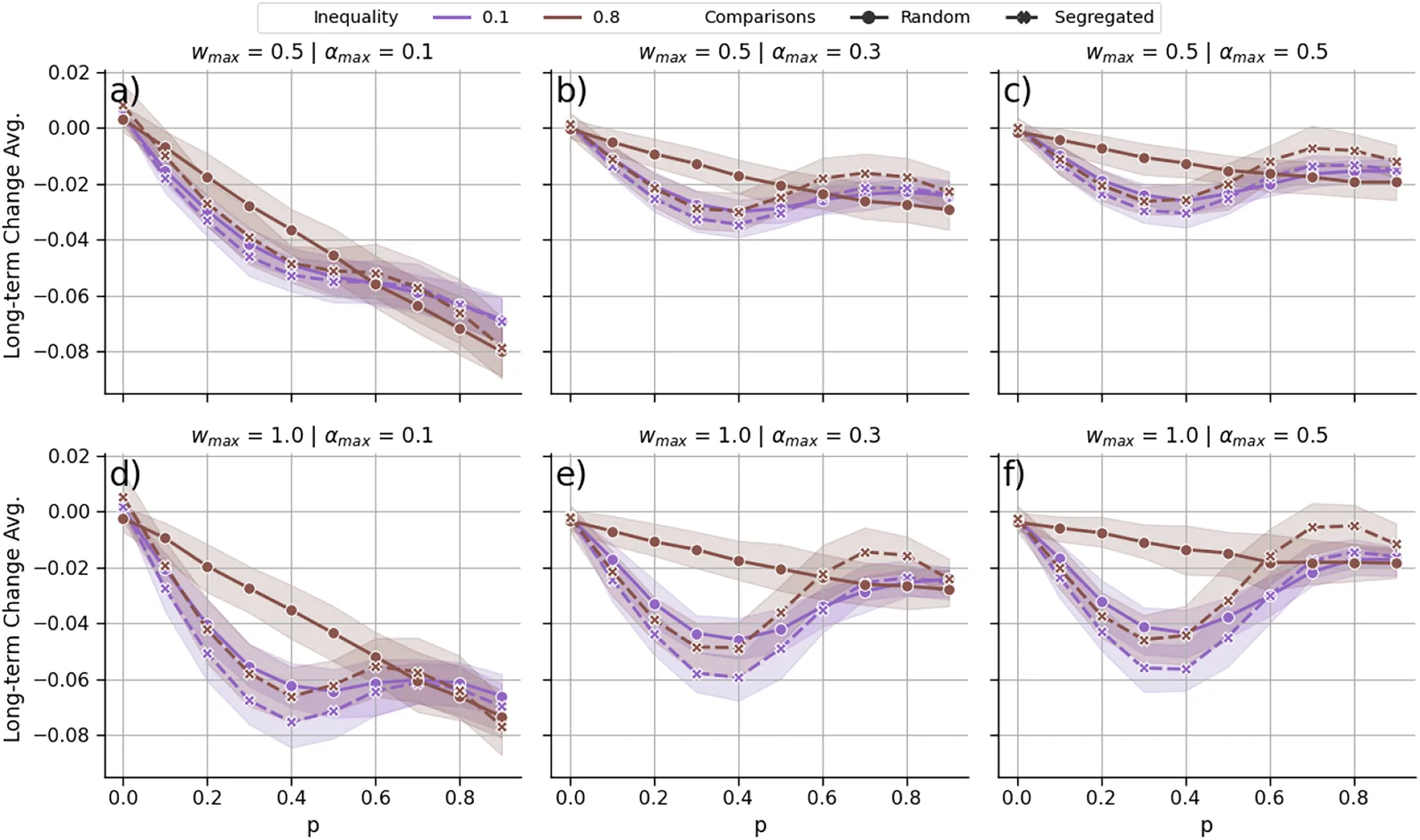
Social normativity. **a** Average long-term change as a function of the share of shocked agents for a maximum social comparison weight of 0.5 and the lowest maximum adaptation rate. **b** Same as (**a**), with an intermediate maximum adaptation rate. c Same as (**a**), with the highest maximum adaptation rate. **d** Average long-term change as a function of the share of shocked agents for a maximum social comparison weight of 1 and the lowest maximum adaptation rate. **e** Same as (**d**), with an intermediate maximum adaptation rate. **f** Same as (**d**), with the highest maximum adaptation rate. Average long-term changes as a function of the percentage of individuals that receives a negative shock of 10,000 in the simulation, two different comparison groups (e.g., random, segregated), the level of inequality (*σ*) and the upper bounds of the adaptation rate (*α*_*max*_) and social comparison weights (*w*_*max*_). The plots are based on 500 agents and 100 replications per parameter configuration and the bounds signify the 5% and 95% percentile. Fixed parameter values can be found in Table 1.

An unexamined parameter in the analysis thus far is the proportion of individuals impacted by a significant negative shock. According to the social normativity hypothesis a shock can be less harmful to SWB when it is prevalent (i.e„ larger percentage affected) due to reduced stigma, shame, and guilt for example (Torche and Daviss, 2025). This hypothesis is also confirmed in most of our simulations, as shown in Fig. 9. Specifically, in all but the random-unequal scenario, an initial increase in the percentage of affected individuals (*p*) leads to a sharp reduction in the average long-term changes in subjective well-being (SWB) and a rise in standard deviation (Supplementary Fig. C7).

The initial rise in variability occurs because in scenarios with low *p*, those impacted by the shock are often isolated within their comparison groups. As a result, their “net change” (Fig. 7) is significantly negative. However, as *p* increases, the likelihood of multiple individuals within the same comparison group being shocked rises, causing the negative impact to level off. When adaptation occurs rapidly (*α*_max_ ≥ 0.2), these effects are less pronounced for *p* ≥ 0.6. Similarly, capping the social comparison weight at 0.5 substantially reduces disparities as *p* increases. This dynamic highlights the dual nature of social comparison. On one hand, it prevents full adaptation to negative events, amplifying the impact of income losses-particularly when few individuals are affected (small *p*). On the other hand, it softens the blow when shocks are widespread, distributing the psychological burden more evenly across comparison groups. Interestingly, the largest long-term SWB changes occur not at *p* = 0.5 but around *p* = 0.4. This can be explained by prospect theory, which emphasizes that losses are perceived as more severe than equivalent gains (Fig. 2). Interestingly, the random-unequal scenario diverges from the social normativity hypothesis. In this case, the average long-term SWB change decreases linearly as *p* increases, suggesting that the protective effects of social comparison are absent in this network structure. These findings underscore the complex interplay between social comparison, network dynamics, and prevalence of shocks in shaping well-being outcomes.

From a welfare perspective, policymakers might not only want to look at average long-term changes in SWB, but also at distributional effects. We therefore calculate the welfare function introduced by Sen (1997) for SWB: $\overline{swb}\cdot (1-Gin{i}_{swb})$, where the first term is average SWB in the society and the second term takes inequality into account, by measure of the Gini coefficient. Figure 6, Supplementary Figs. C2, C6 show that this measure does not qualitatively deviate from the average long-term changes in SWB and therefore would not lead to differences in policy recommendations.

#### Subjective well-being traps

**Fig. 10:**
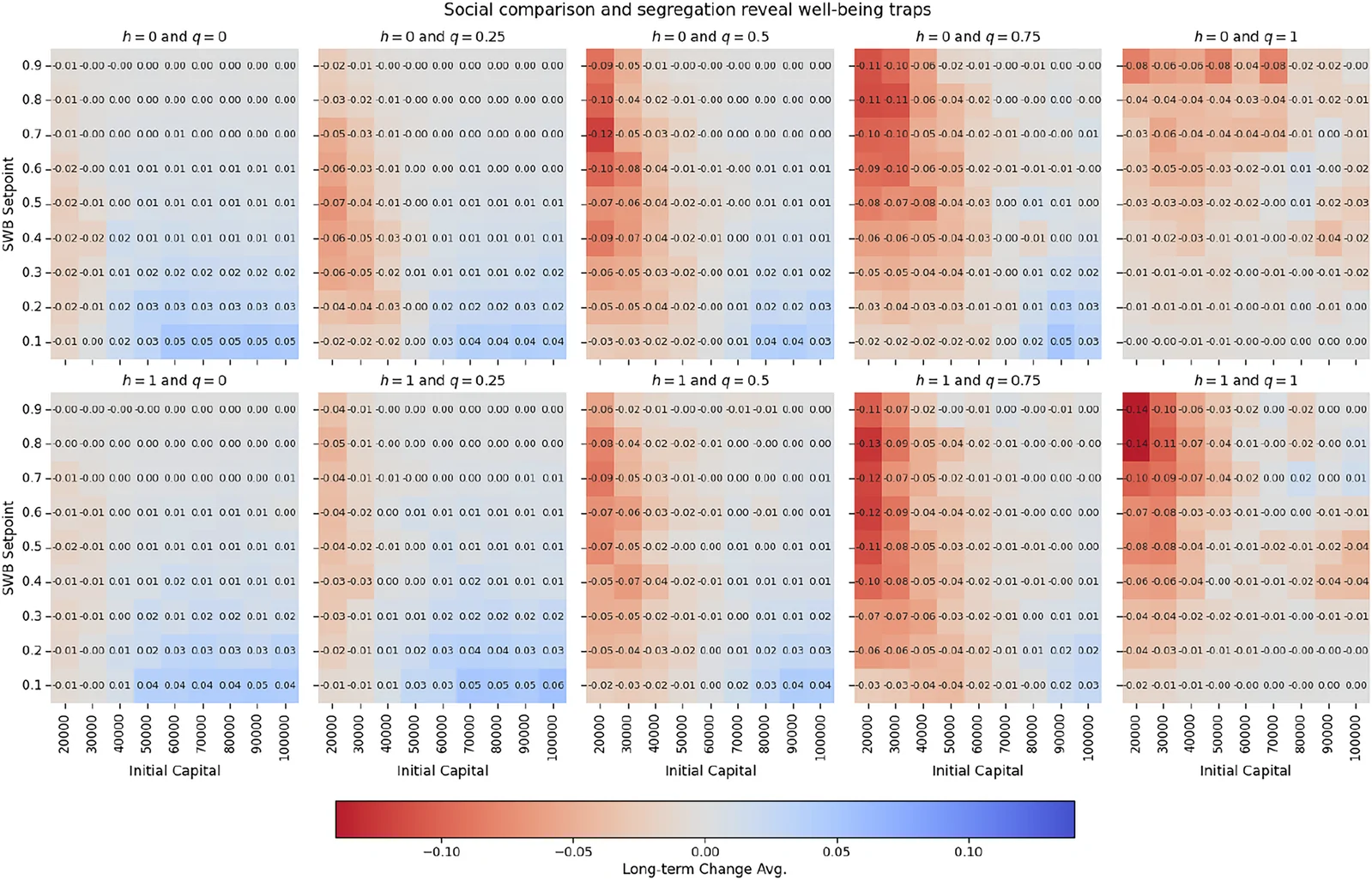
Subjective well-being traps. Every cell in the heatmap represents the average change in SWB (*t* = 0 to *t* = 99) calculated over 30 simulations for individual *j*. Individual *j* is given the initial income and SWB set-point that belong to the specific cell and in addition: *α*_*j*_ = 0.2, *δ*_*j*_ = 1, *w*_*j*_ = 0.5. The rest of the individuals have parameter values according to the fixed values in Table 1 in a relatively unequal society (*σ* = 0.8). In addition, 50% of the society + individual *j* gets the large negative shock.

To analyse which individuals are the most affected in these artificial societies, Fig. 10 shows the change in SWB for individuals with a specific set-point and initial income. More specifically, every cell in the heatmap represents the average change in SWB (*t* = 0 to *t* = 99) calculated over 30 simulations for individual *j*. Individual *j* is given the initial income and SWB set-point connected to the specific cell and, in addition: *α*_*j*_ = 0.2, *δ*_*j*_ = 2, *w*_*j*_ = 0.5. The simulations start with *σ* = 0.8 and initial income thus is relatively unequally distributed. Individuals other than *j* have parameter values according to the fixed values in Table 1. Furthermore, 50% of the society + individual *j* receives the large negative shock. It should also be noted that this change in SWB is a different calculation than the long-term change of the previous sections, as it takes the set-point into account and not average SWB from *t* = 30 to *t* = 49 per individual. Hence, the changes in adaptation levels because of initial differences in income are also reflected here. For an illustration, see the difference between random and segregated comparison in the initial time steps of Supplementary Fig. C1.

Figure 10 highlights that individuals with relatively high income but low set points of SWB (*swb*_*i*(*t*=0)_ ≥0.4) are more likely to experience long-term positive changes in SWB, especially when the society compares to lower quantiles (*q* ≤ 0.5). Such individuals are likely to compare downward and thus perceiving their income values more positively. These effects are more substantial for random comparison groups than for segregated, because the differences in income are greater in the random network. Moreover, although these agents are negatively shocked at *t* = 50 they are able to recover or even improve their SWB when *q* < 1. When *q* = 0 and everyone is comparing to the minimum in their reference group, full recovery is possible even with low income. However, these individuals are essentially trapped in their initial SWB (i.e., set-point) as they are comparing upward and not improving their SWB such as their richer counterparts. Here we loosely define being trapped as not being able to move out of one’s initial state of SWB or not fully recovering from severe shocks. Furthermore, when the society compares to the median (*q* = 0.5) or higher, individuals are unable to fully adapt, and this effect is most severe for those with low initial income but a high set-point. The former is because they compare upward due to their low income and the latter because of the sigmoid function (Eq. (1)). Losses are felt more strongly than gains and the sigmoid is steeper (in case of income losses) for a person with a set-point of 0.9 versus an individual with 0.1. Thus, those with high set-points but low income are most at risk of being trapped and not fully recovering of substantial shocks to their income level. Overall, these effects are exacerbated in random comparison groups because of larger income variability, and hence individuals are more protected (and stable) when comparison groups are segregated with respect to income.

As a robustness check, a different functional form of the SWB function (Supplementary Fig. D1) is implemented. More specifically, the difference between income and adaptation level is now transformed using a square root function instead of the sigmoid of Eq. (1). Using this concave function, we reran the well-being traps experiment and show that the traps remain (Supplementary Fig. D4) and are therefore not dependent on the shape of the SWB function.

## Discussion

This study presents an agent-based model (ABM) that tries to disentangle how subjective well-being (SWB) is affected by the complex interplay of individual characteristics, social aspects, and environmental factors that together form a multidimensional space of nonlinear interactions, including feedback loops, and temporal effects. The model incorporates interactions between heterogeneous individuals that adapt to changes in their income and compare themselves with others through social structures. Isolated individuals adhere to the adaptation theory (Brickman, 1971), such that changes in income lead to complete adaptation over time through habituation and adjustment processes. Based on the revised adaptation theory (Diener et al., 2009), the model further accounts for individual variability in adaptation rates, SWB set-points, emphasis on social comparison, and the degree of SWB feeding back into income. This model allows us to explore a number of complex dynamics that are important to SWB. The model generates four key insights.

First, the model shows how social comparison can have long-term effects on SWB after significant economic shocks, such as unemployment crises or natural disasters. Thus, although individuals would fully adapt due to adaptation theory, social comparison can obstruct this. Importantly, most individuals demonstrate considerable resilience, (almost) fully adapting to changes. This finding is consistent with a systematic review that highlights resilience as the predominant response trajectory, even under substantial stress (Galatzer-Levy et al., 2018). However, when individuals strongly emphasise social comparison and the shock changes the income of the individual relative to its comparison group, long-term changes in SWB can be substantial. Two primary mechanisms underlie this long-term influence of social comparison: (a) Income Variability and Inequality Amplification: High variability in the income of comparison groups, particularly when combined with pronounced multiplicative income growth, intensifies inequalities over time, exerting a sustained impact on SWB. (b) Impact of Segregation and Isolated Shocks: Even in contexts with low income variability (e.g., segregated networks), a significant shock to an individual’s income can lead to lasting changes in SWB, especially if others in their comparison group remain unaffected by the shock.

Second, when individuals engage in downward comparisons, the average long-term impact on SWB is positive, and individuals fully adapt to even substantial shocks. In contrast, upward comparisons lead to reduced long-term SWB and increased volatility at the population level. This aligns with previous research indicating that upward social comparisons are the norm and often result in dissatisfaction rather than motivation for improvement (Gerber et al., 2018). The quantile used for comparisons (i.e. *q*), effectively changes the fraction of downward/upward comparisons, and hence a higher *q* results in more pronounced negative effects for both individuals and the population.

Third, social comparison can act as a double-edged sword: Certain individuals do not fully adapt, as described in the previous sections, and, especially when only a few individuals are affected, their SWB can change substantially. However, social comparison also reduces the effect on SWB when a very large fraction of the population is shocked, dampening the effects as many individuals in the comparison group are also affected. Evidence for this social normativity hypothesis is also found in recent empirical work (Torche and Daviss, 2025).

Lastly, especially individuals with low (initial) income and moderate to high SWB set-points are at risk of long-term changes in SWB in our simulations. Hence, such individuals might be of interest for more targeted interventions and/or prevention. However, segregated comparison groups mitigate this effect to some degree, as the results indicate that segregated networks demonstrate less pronounced changes in SWB, even when individuals engage in upward social comparisons. This stability can be attributed to lower income variability in the comparison groups (i.e., segregation) which reduces the impact of social comparison.

When mapping real-world societies onto the model, the most realistic configuration aligns with the unequal and segregated scenario (McPherson et al., 2001). While segregated comparison groups may foster greater stability and moderate the impact of shocks on subjective well-being (SWB), inequality introduces significant income variability, intensifying the effects, particularly when combined with multiplicative income growth. Furthermore, in some countries, less affluent individuals experience lower income growth compared to their wealthier counterparts (Baggio and Papyrakis, 2014). This disparity exacerbates the negative effects of social comparison, as those with lower income struggle to keep pace. Additionally, upward social comparisons are the norm (Gerber et al., 2018), making higher values for *q* more plausible. In our simulations, these dynamics result in substantial long-term changes in SWB at both the individual and societal levels, underscoring the profound influence of inequality and comparison on well-being outcomes.

## Limitations and further research

Drawing from separate theoretical and empirical components, the modelling allows us to integrate different aspects that together constitute the internal validity of the model. Inevitably, there is an element of self-confirmation. The combination of existing components on the basis of a number of plausible assumptions also means that the model cannot do anything other than confirm that the assumptions are plausible. Although some aspects are verified against the survey materials, the external validity must be strengthened. For instance, the implemented mechanisms for social comparison and adaptation could be validated more (i.e., on the micro-level), but also relationships and hypotheses that emerge on the macro-level can be tested and validated (see Bruno (2025) for an example).

In line with our main findings, four hypotheses can be formulated and tested: (H1) Individuals who frequently engage in upward social comparisons experience a lower long-term subjective well-being (SWB) than those who engage in downward comparisons. (H2) The negative impact of economic shocks on SWB is greater in individuals with low income and high SWB set-points, compared to others. (H3) In societies with higher levels of social inequality and segregation, the buffering effect of social comparison during widespread economic shocks is stronger than in more integrated societies. (H4) SWB adaptation to income shocks is incomplete in the presence of strong social comparison dynamics, especially when individuals are sensitive to others’ incomes.

As with any model, simplifying assumptions are made. This is inevitable given the complexity of the topic. As mentioned above, no single study can capture all aspects related to SWB. For example, the model does not consider the effects of other environmental conditions on SWB, such as architecture and the built environment (Mazumdar et al., 2018; Mouratidis, 2021), green spaces (Hunter et al., 2019), or air pollution (Andrei et al., 2024). Similarly, personality characteristics can be further deepened. Other types of factors may also play a role, such as social income (Ehsan et al., 2019). In addition, personality, adaptation rates, income, and social comparison weights are all modelled as independent, while it is very likely that many dependencies between these factors exist.

## Supplementary information

Supplementary Information

## Data Availability

The data that support the findings of this study are available from https://www.lissdata.nl, but restrictions apply to the availability of these data, which were used under licence for the current study, and so are not publicly available. Data are, however, available from the authors upon reasonable request and with permission of the LISS Panel.

## Footnotes

1 SWB is often divided into components such as life satisfaction and positive/negative affect; however, this study uses SWB as a unified construct.

2 Sociologically, the term “segregation” might carry specific connotations tied to structural, institutional, or policy-driven mechanisms that intentionally or unintentionally separate groups. “Choice homophily” is about individuals’ preferences and tendencies to associate with similar others. In this study we focus on the effects of segregation, irrespective of its underlying mechanism, on the behaviour of the model.

3 It should be noted that this correlation is substantially lower in the segregated network (Supplementary Fig. A3).

4 Their definition of resilience is “the maintenance or fast recovery of mental health during or after stressor exposure”.

## Code availability

Code to reproduce the simulations and analyses can be found here: https://github.com/ericdignum/wellbeing-abm/.
