# Supplementary material for: Social comparison and segregation reveal well-being traps
Source: Humanit Soc Sci Commun. 2026 Jun 11;13(1):1625. doi: 10.1057/s41599-026-07875-9 (PMC13612229; doi:10.1057/s41599-026-07875-9)
Supplement: Supplementary file 1 [file 41599_2026_7875_MOESM1_ESM.pdf]

Supplementary information: Social comparison and segregation reveal well-being traps

Appendix A: Additional model results

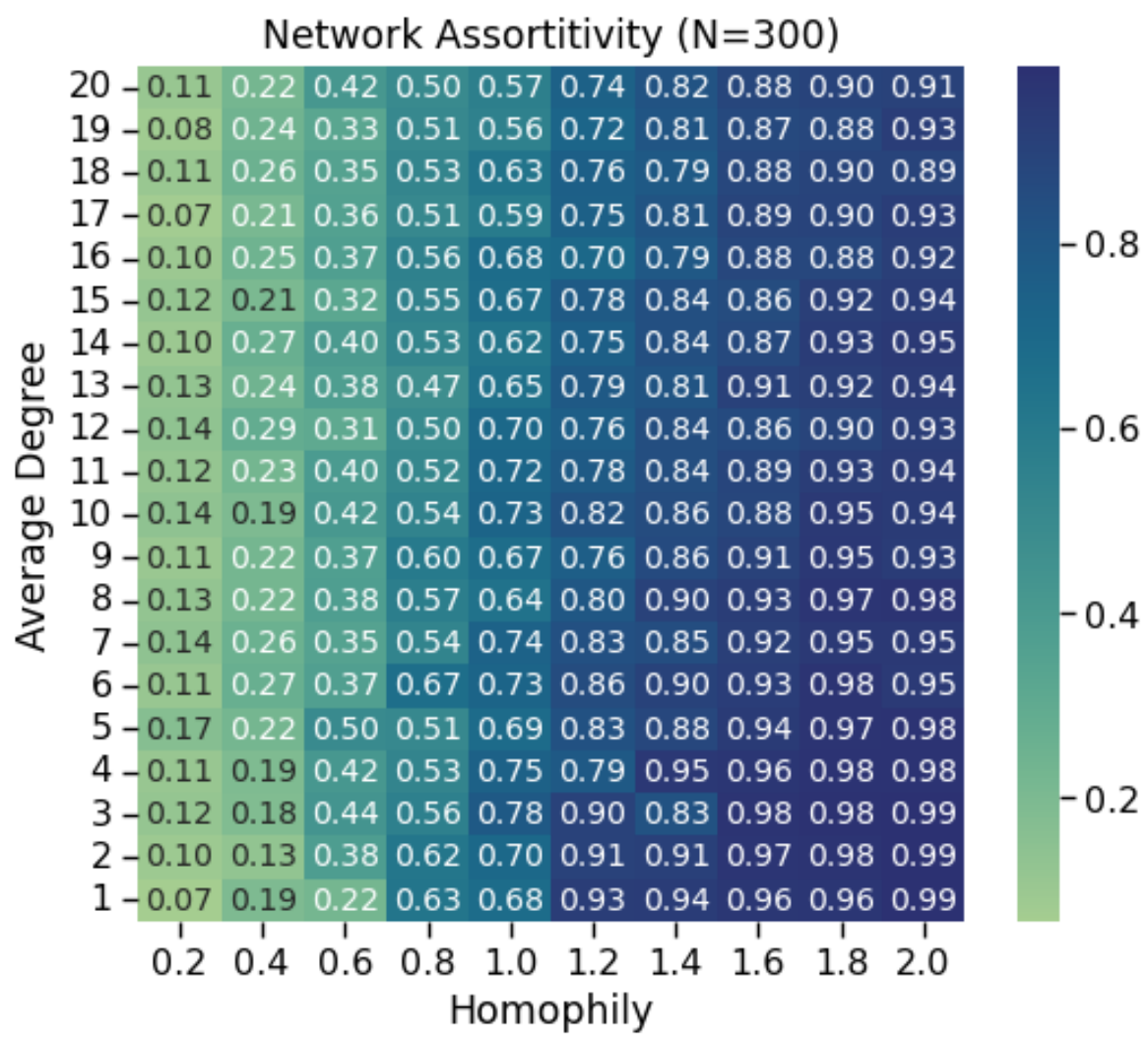

Fig. A1: Assortitivity for networks with different levels of homophily and average degree ($N = 500$). Values are based on one realisation of the network for a given parameter combination.

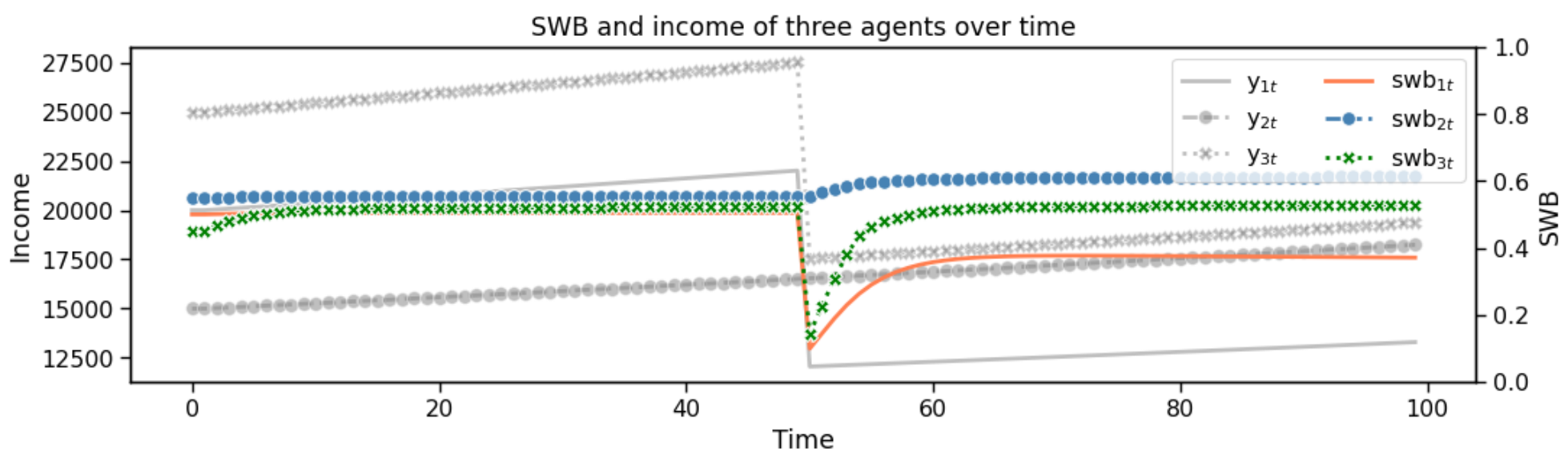

Fig. A2: A simulated society with three agents. Agent 1 starts with a set-point of 0.5 (solid orange line) and income of 20000, Agent 2 with 0.55 and 15000, and Agent 3 with 0.45 and 25000. Agent 1 socially compares to 2 and 3, while 2 and 3 only compare to 1 ($w_1 = w_2 = w_3 = 0.5$). Agent 1 and 3 receive a shock of -10,000 at $t = 50$. There is no stochasticity in the income. Agent 3 receives a shock, but fully habituates as it is comparing to Agent 1 who also had a shock. However, Agent 1 does not fully adjust to the shock, as it transitions from a mid-ranking position in terms of income to the lowest rank, thereby altering its comparative value. Additionally, Agent 2 receives a lasting positive change because of this switch in comparisons.

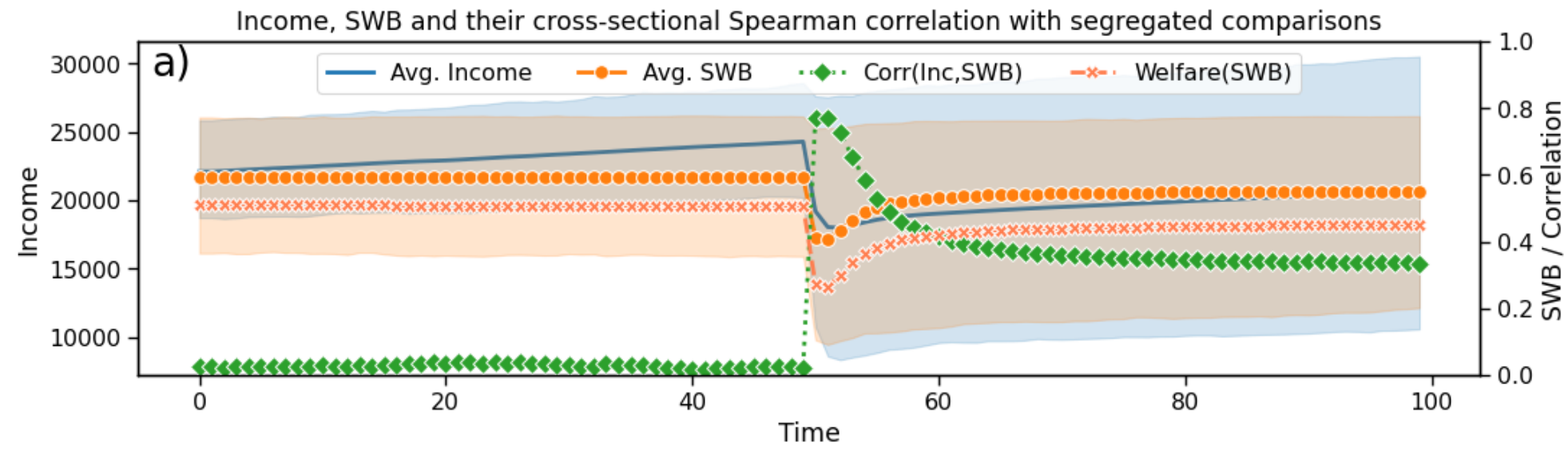

Fig. A3: Well-being, income and their correlation over time when comparing on a **segregated** network. Bounds indicate the 5% and 95% percentile and 50% of the individuals receive a negative shock at $t = 50$. Fixed parameter values can be found in Table 1. Simulations are with $N = 500$.

Appendix B: Global sensitivity analysis

For global sensitivity analysis, PAWN sensitivity analysis (Pianosi & Wagener, 2015, 2018) is applied to identify influential input parameters on the specific outputs. Values for the input parameters are sampled according to the bounds stated in Table 1 and a Sobol sequence with sample size 8192, resulting in $\tilde{1}80{,}000$ simulations in total. The PAWN method compares the empirical cumulative distribution functions of model outputs when an input is fixed versus when it is varied, using the median Kolmogorov–Smirnov statistic as the sensitivity index. Contrary to the frequently used Sobol method for sensitivity analysis, PAWN takes the whole cumulative distribution into account and thus can handle more complicated output distributions.

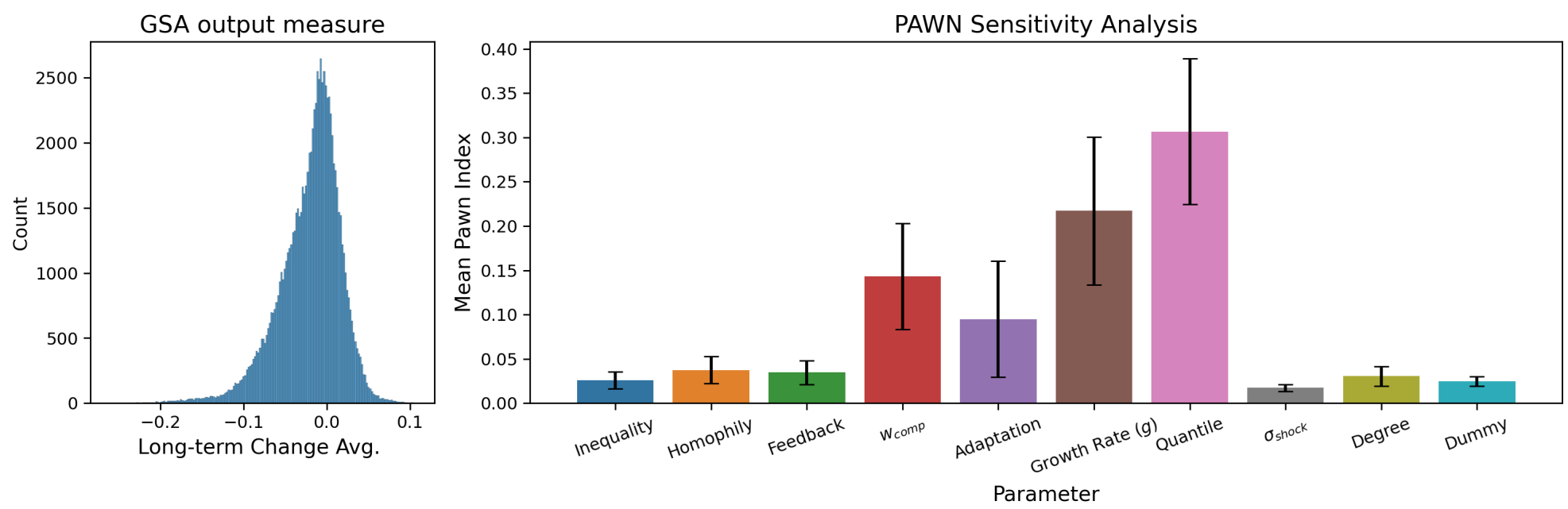

Fig. B1: PAWN (median) sensitivity analysis of the average long-term change in SWB. Bounds for the parameters can be found in Table 1. Plots are based on a Sobol sequence with sample size of 4096, resulting in 90112 simulations in total.

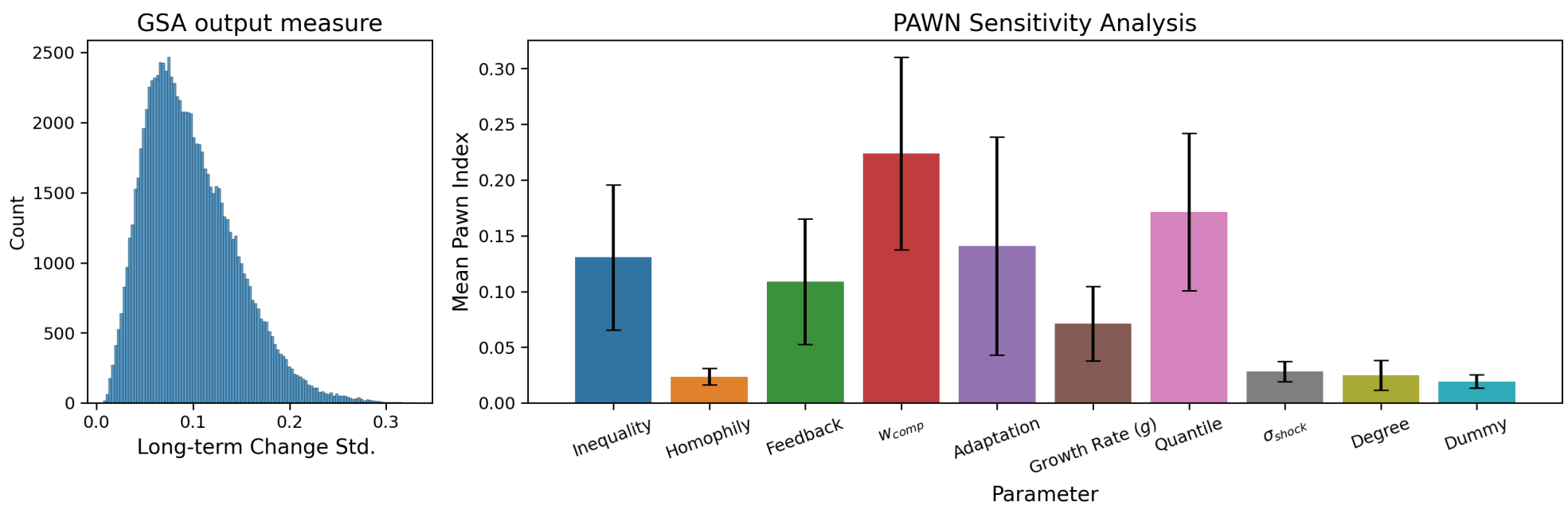

Fig. B2: PAWN (median) sensitivity analysis of the standard deviation of the long-term change in SWB. Bounds for the parameters can be found in Table 1. Plots are based on a Sobol sequence with sample size of 4096, resulting in 90112 simulations in total.

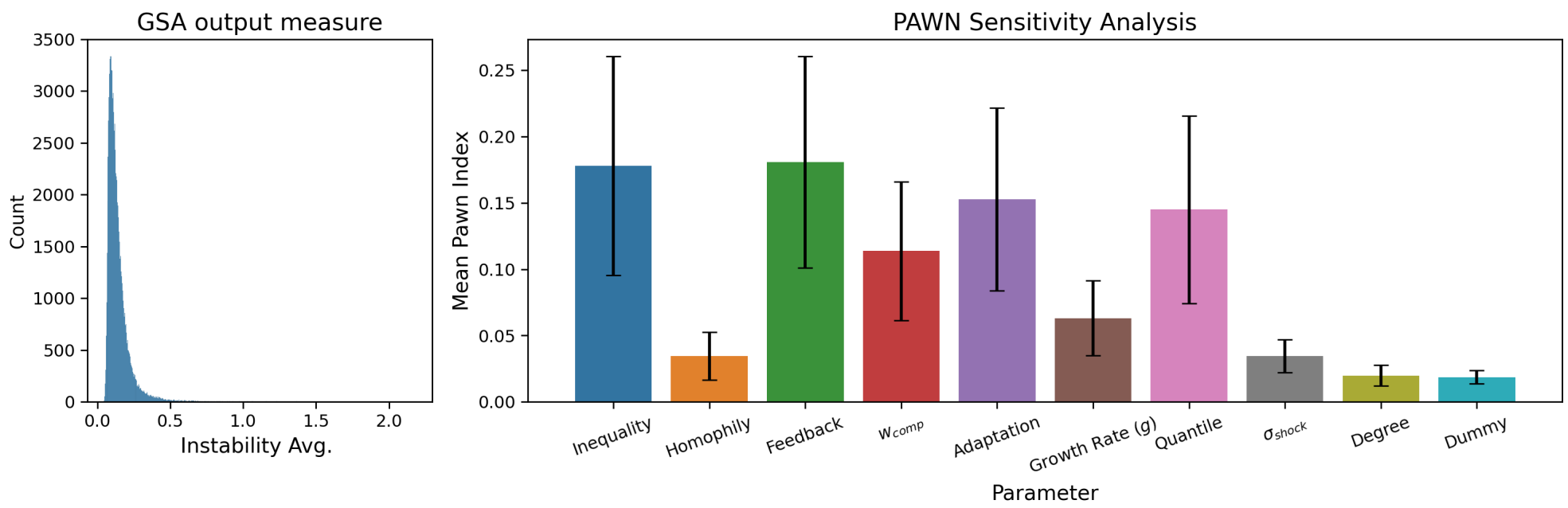

Fig. B3: PAWN (median) sensitivity analysis of the instability in SWB. Bounds for the parameters can be found in Table 1. Plots are based on a Sobol sequence with sample size of 4096, resulting in 90112 simulations in total.

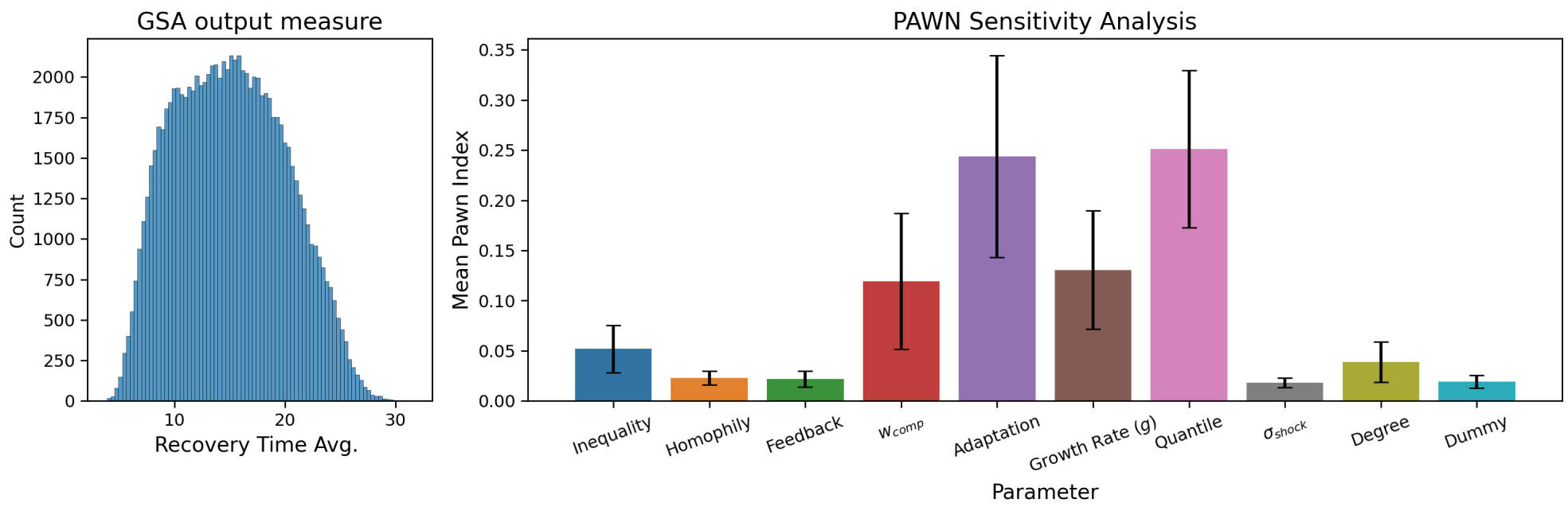

Fig. B4: PAWN (median) sensitivity analysis of the recovery time. Bounds for the parameters can be found in Table 1. Plots are based on a Sobol sequence with sample size of 4096, resulting in 90112 simulations in total.

Appendix C: Additional experimental results

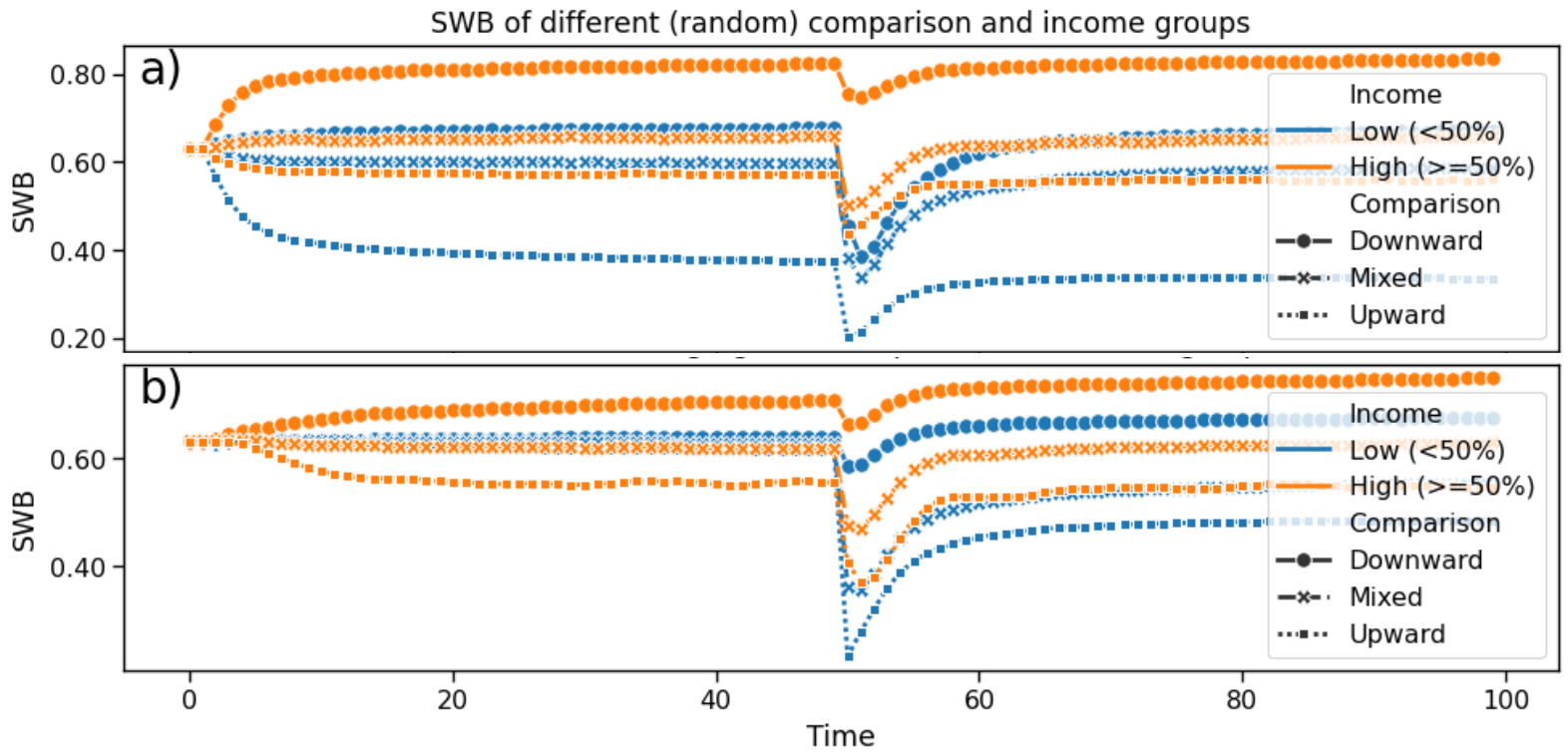

Fig. C1: **Comparison groups.** An unequal ($\sigma = 0.8$) simulated society ($N = 500$) where individuals have on average 10 connections in a random (a) or segregated (b) network and compare themselves to the 50% percentile ($q = 0.5$). All agents receive small perturbations $\sim N(g = 0.002, \sigma_{shock} = 0.005)$ in every time step. Other fixed parameter values can be found in Table 1. Six different groups are plotted. Those who compare upward at least 90% of the simulation time, i.e., their income is lower than the quantile they compare to for at least 90 out of 100 time steps. Similarly, agents that compare downward 90% of the time and a mixed group that does not fall in any of the other two. In addition, in every time step, 50% of the individuals with the lowest income (blue) and the 50% of the highest are separated out. Only averages (based on 100 repetitions) are plotted to reduce clutter.

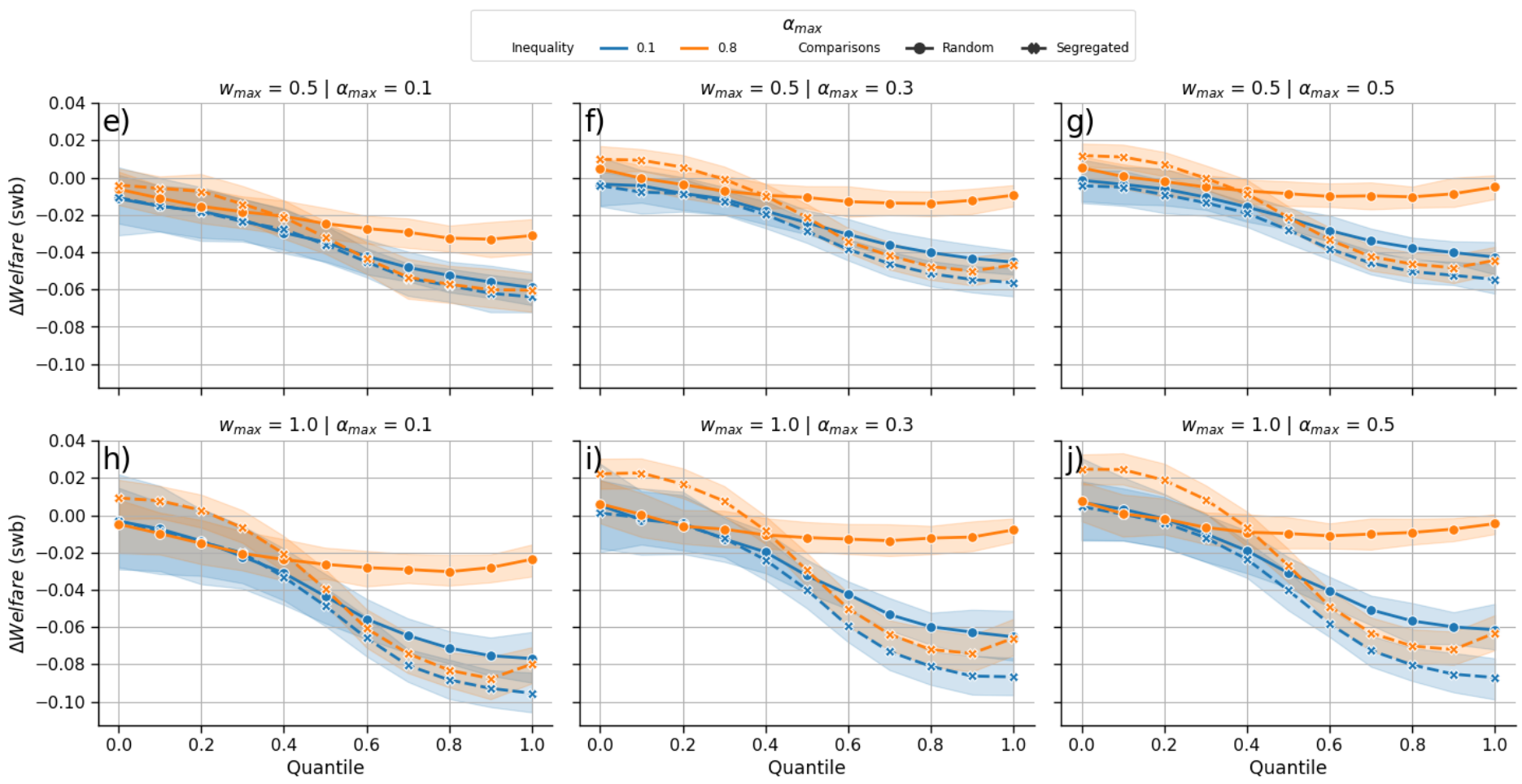

Fig. C2: Welfare as a function of the quantile one compares to, two different network structures (e.g., random, segregated), the income growth rate (g) and the upper bounds of the adaptation rate (α_{max}) and social comparison weights (w_{max}). The plots are based on 500 agents and 100 replications per parameter configuration and the bounds signify the 5% and 95% percentile. Fixed parameter values can be found in Table 1.

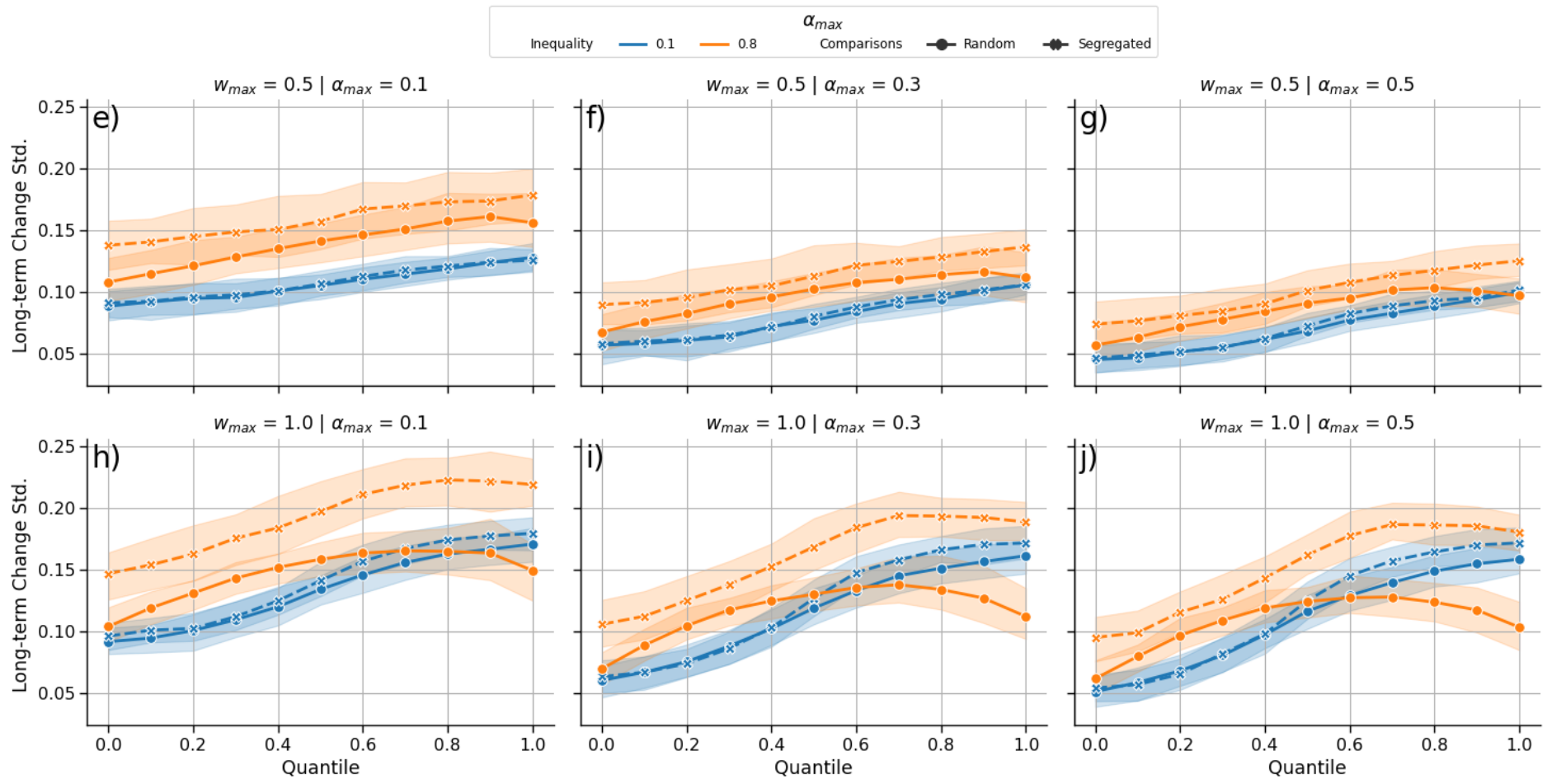

Fig. C3: Standard deviation of long-term changes as a function of the quantile one compares to, two different network structures (e.g., random, segregated), the income growth rate (g) and the upper bounds of the adaptation rate (α_{max}) and social comparison weights (w_{max}). The plots are based on 500 agents and 100 replications per parameter configuration and the bounds signify the 5% and 95% percentile. Fixed parameter values can be found in Table 1.

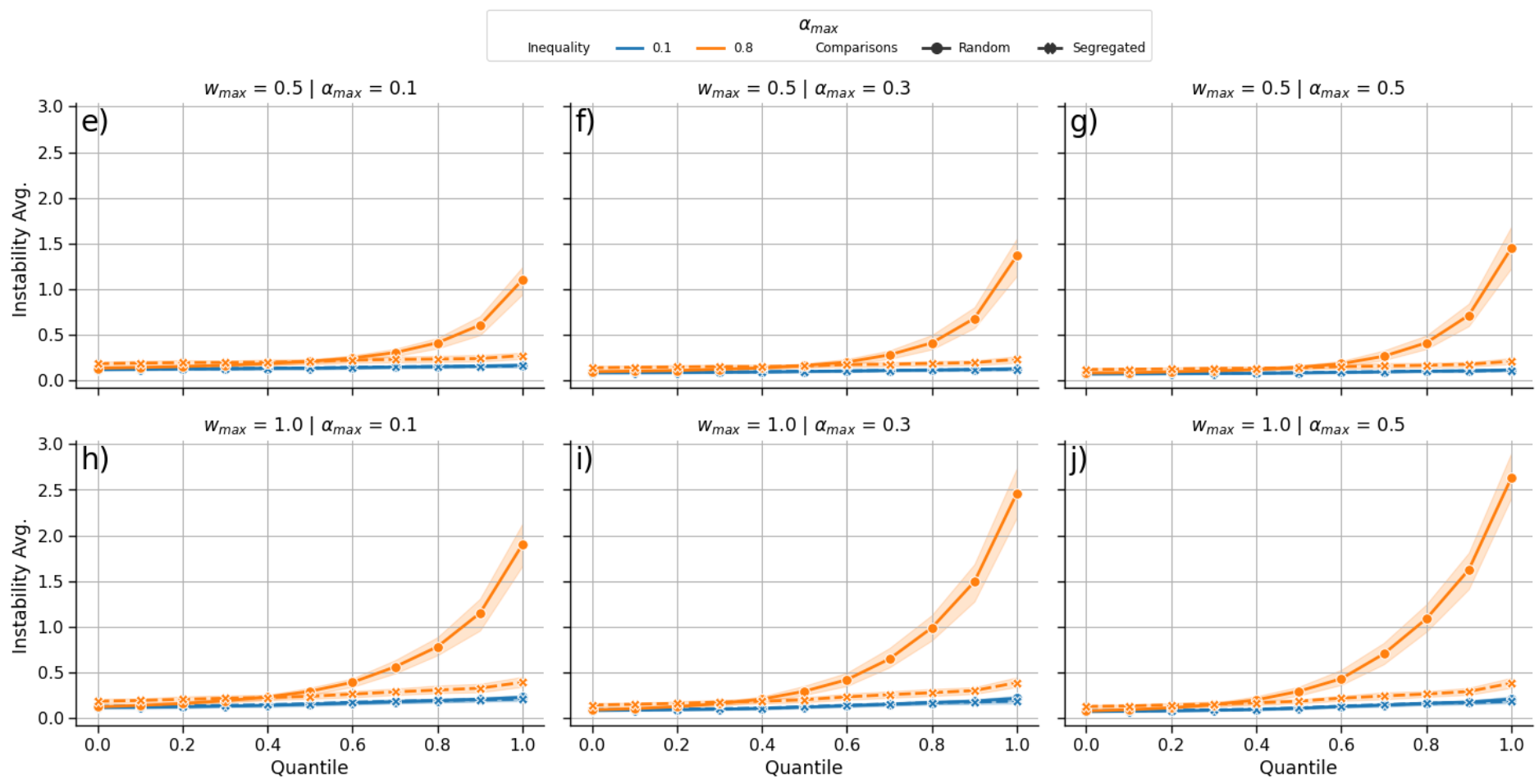

Fig. C4: Instability as a function of the quantile one compares to, two different network structures (e.g., random, segregated), the income growth rate (g) and the upper bounds of the adaptation rate (α_{max}) and social comparison weights (w_{max}). The plots are based on 500 agents and 100 replications per parameter configuration and the bounds signify the 5% and 95% percentile. Fixed parameter values can be found in Table 1.

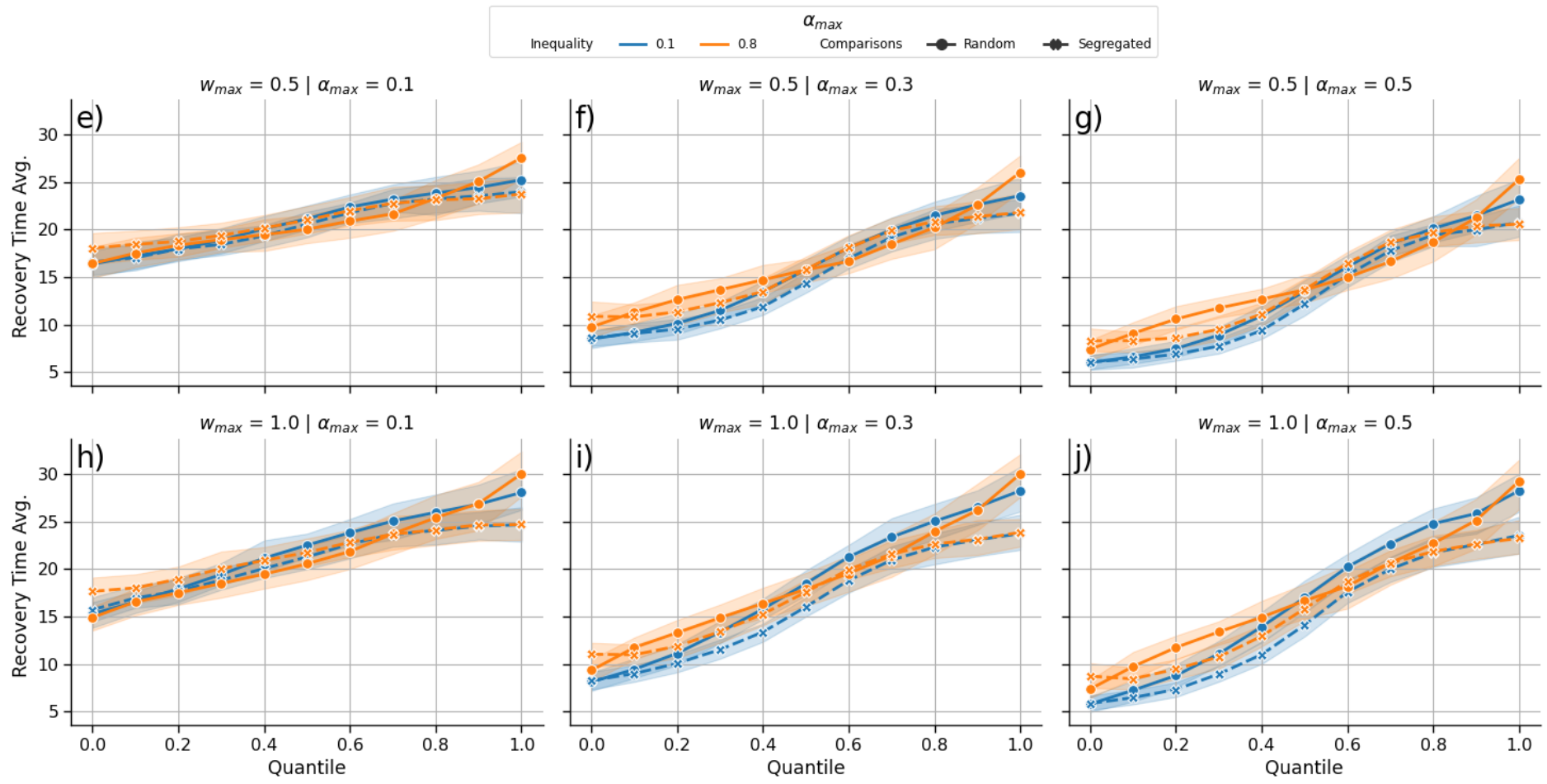

Fig. C5: Recovery times as a function of the quantile one compares to, two different network structures (e.g., random, segregated), the income growth rate (g) and the upper bounds of the adaptation rate (α_{max}) and social comparison weights (w_{max}). The plots are based on 500 agents and 100 replications per parameter configuration and the bounds signify the 5% and 95% percentile. Fixed parameter values can be found in Table 1.

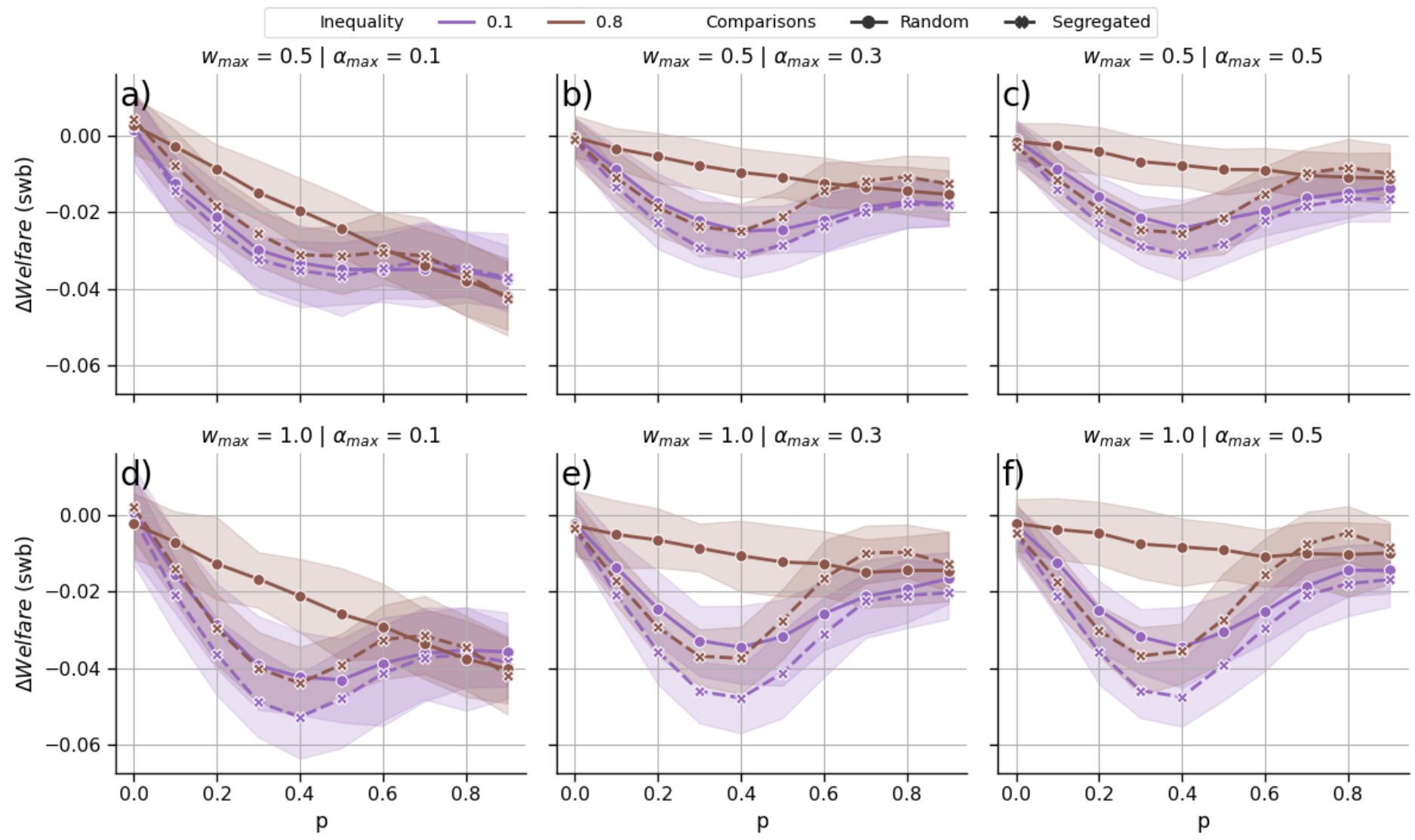

Fig. C6: **Social normativity.** Welfare as a function of the percentage of individuals that receives a negative shock of 10,000 in the simulation. Two types of comparison groups (e.g., random, segregated), the income growth rate (g) and the upper bounds of the adaptation rate (α_{max}) and social comparison weights (w_{max}). The plots are based on 500 agents and 100 replications per parameter configuration and the bounds signify the 5% and 95% percentile. Fixed parameter values can be found in Table 1.

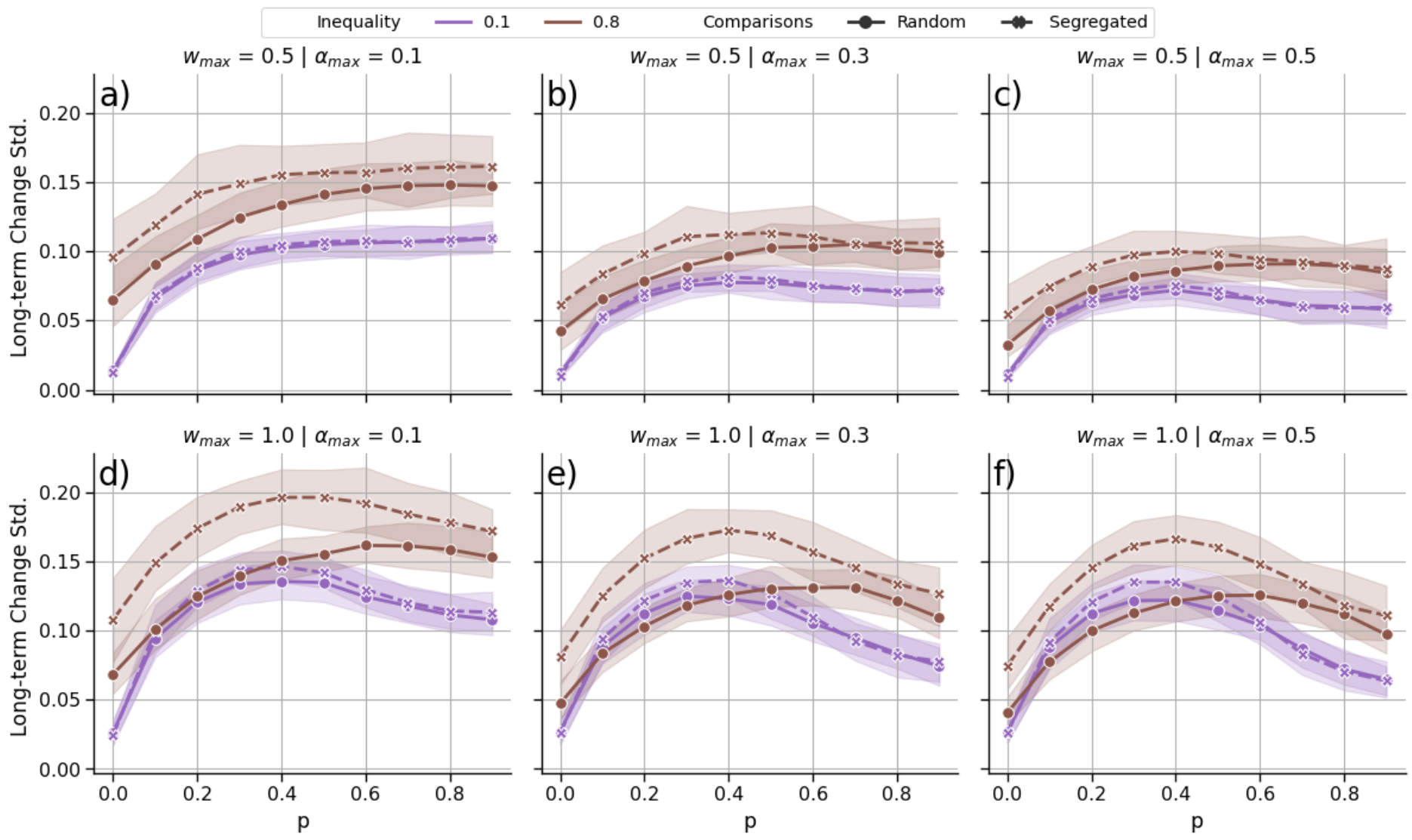

Fig. C7: **Social normativity.** Standard deviation of long-term changes as a function of the percentage of individuals that receives a negative shock of 10,000 in the simulation. Two types of comparison groups (e.g., random, segregated), the income growth rate (g) and the upper bounds of the adaptation rate (α_{max}) and social comparison weights (w_{max}). The plots are based on 500 agents and 100 replications per parameter configuration and the bounds signify the 5% and 95% percentile. Fixed parameter values can be found in Table 1.

Appendix D: Well-being traps experiment with concave SWB function

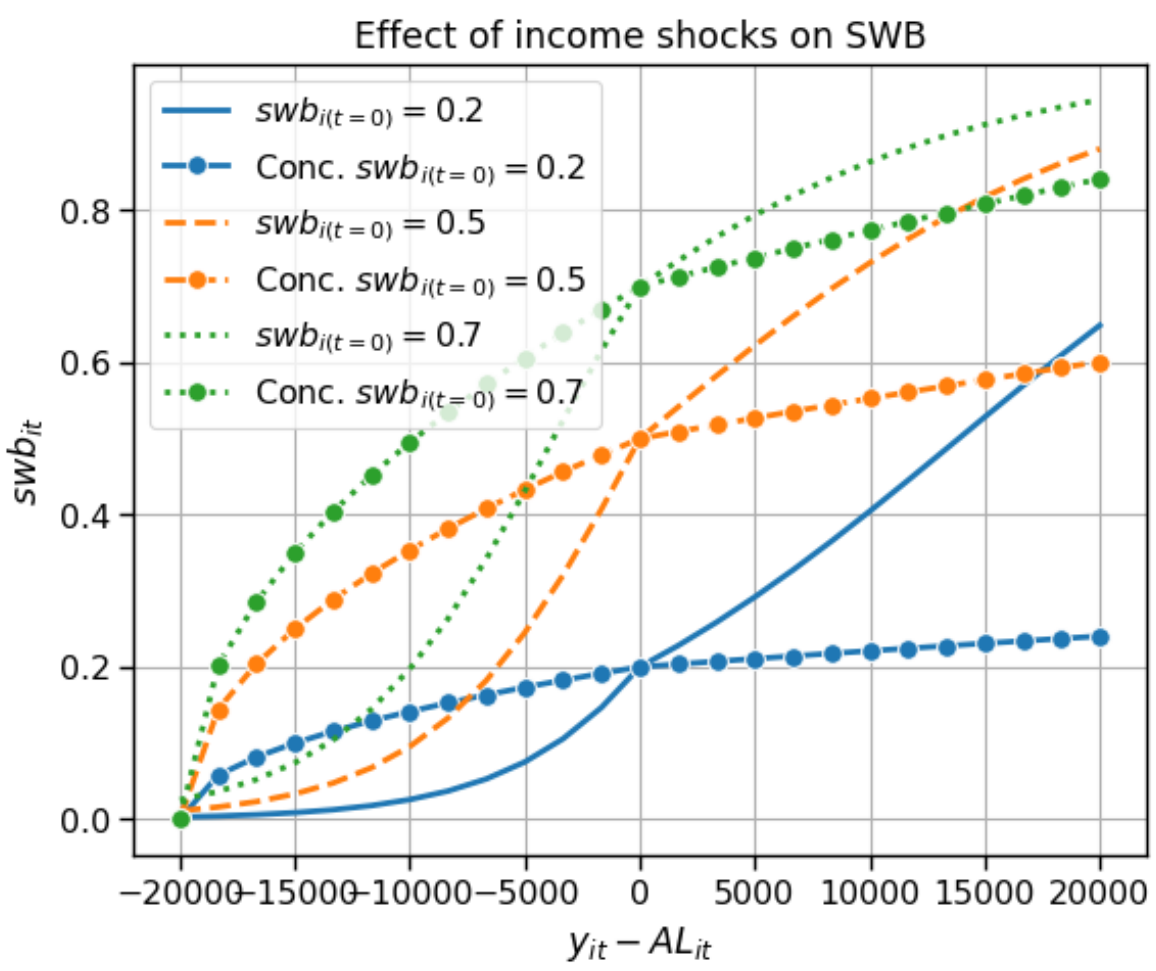

Fig. D1: Concave function used for the experiment of Figure D2: (min $\left\{1, \max\left\{0, \frac{swb_{i(t=0)}}{\sqrt{20000}}\sqrt{y_{it} - AL_{it} + 20000}\right\}\right\}$) as opposed to the sigmoid for different set-points ($swb_{i(t=0)}$). Losses are perceived 2.25 more than gains in accordance with Prospect Theory (Tversky & Kahneman, 1992).

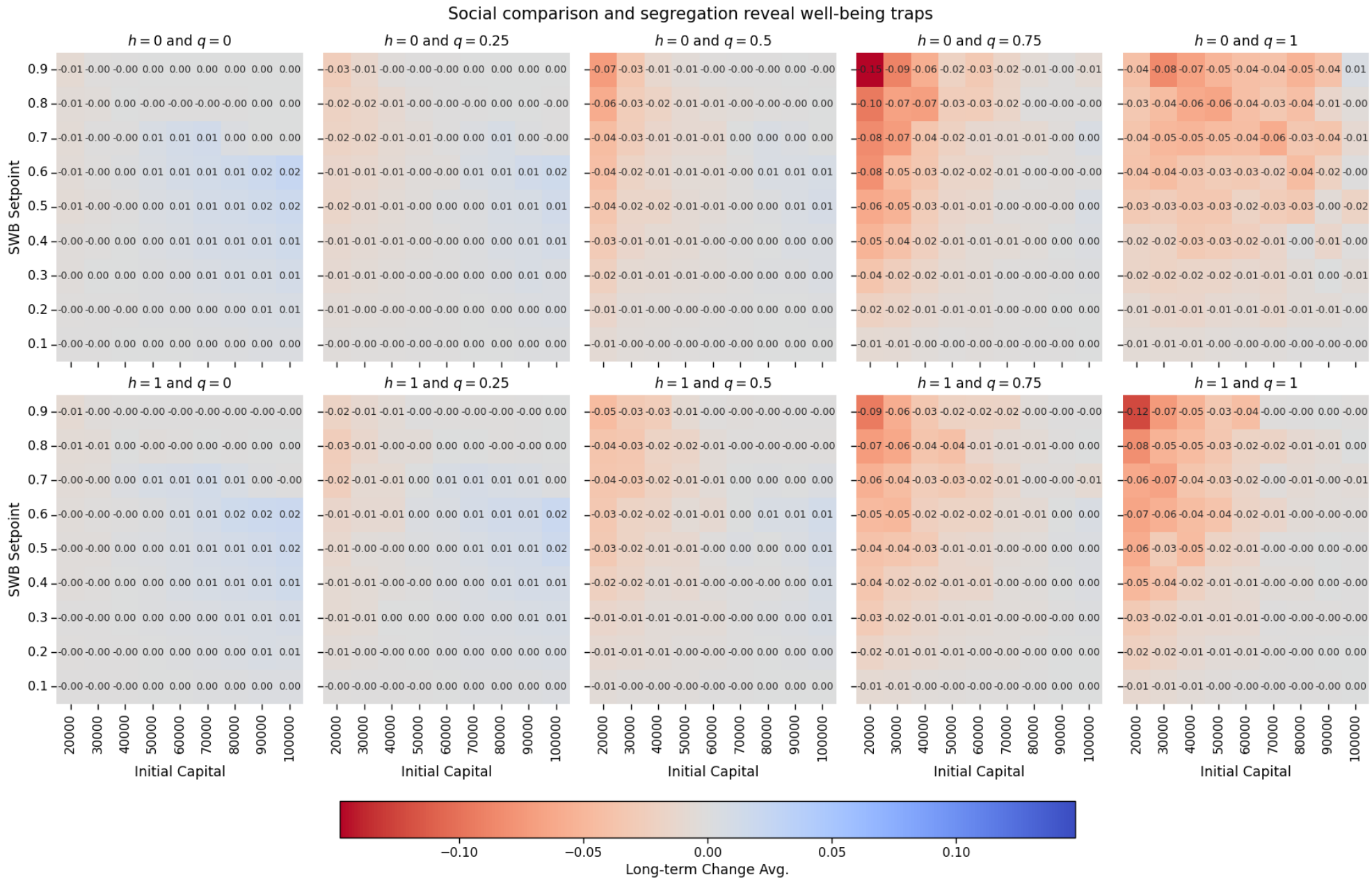

Fig. D2: **Subjective well-being traps with concave function.** Every cell in the heatmap represents the average change in SWB ($t = 0$ to $t = 99$) calculated over 30 simulations for individual j. Individual j is given the initial income and SWB set-point that belong to the specific cell and in addition: $\alpha_j = 0.2, \delta_j = 1, w_j = 0.5$. The rest of the individuals have parameter values according to the fixed values in Table 1 in a relatively unequal society ($\sigma = 0.8$). In addition, 50% of the society + individual j gets the large negative shock.

Appendix E: Validation of agent behaviour

For validation, we make use of data from the LISS panel (Longitudinal Internet studies for the Social Sciences) (Scherpenzeel, 2018), which is administered and managed by the non-profit research institute Centerdata (Tilburg University, the Netherlands). We analyse data from two single-wave studies: one on social comparison and one on neighbourhood perceptions. The former already contained questions on income and life satisfaction, while for the latter we had to combine it with the income domain of the LISS Core Study, which is conducted every year since 2007.

Within the first part of the social comparison survey, respondents were asked in which of seven income buckets their personal net income falls. They were also asked to estimate the percentage of adults in the Netherlands that fall within each income bucket. Rather than the actual income distribution, this gives us an understanding of the perception of relative income among the respondents. The stated estimates are likely informed by the social environment as indicated by Galesic, Olsson, and Rieskamp (2018). Hence, this is a suitable proxy for subjective comparisons on social networks, similar to our model. We then proceed to calculate the median of these estimated distributions (using the midpoints of the buckets) and the difference with their own bucket midpoint. The same survey asks about respondents' life and income satisfaction in the last month, providing us with the cognitive component of SWB with the former.

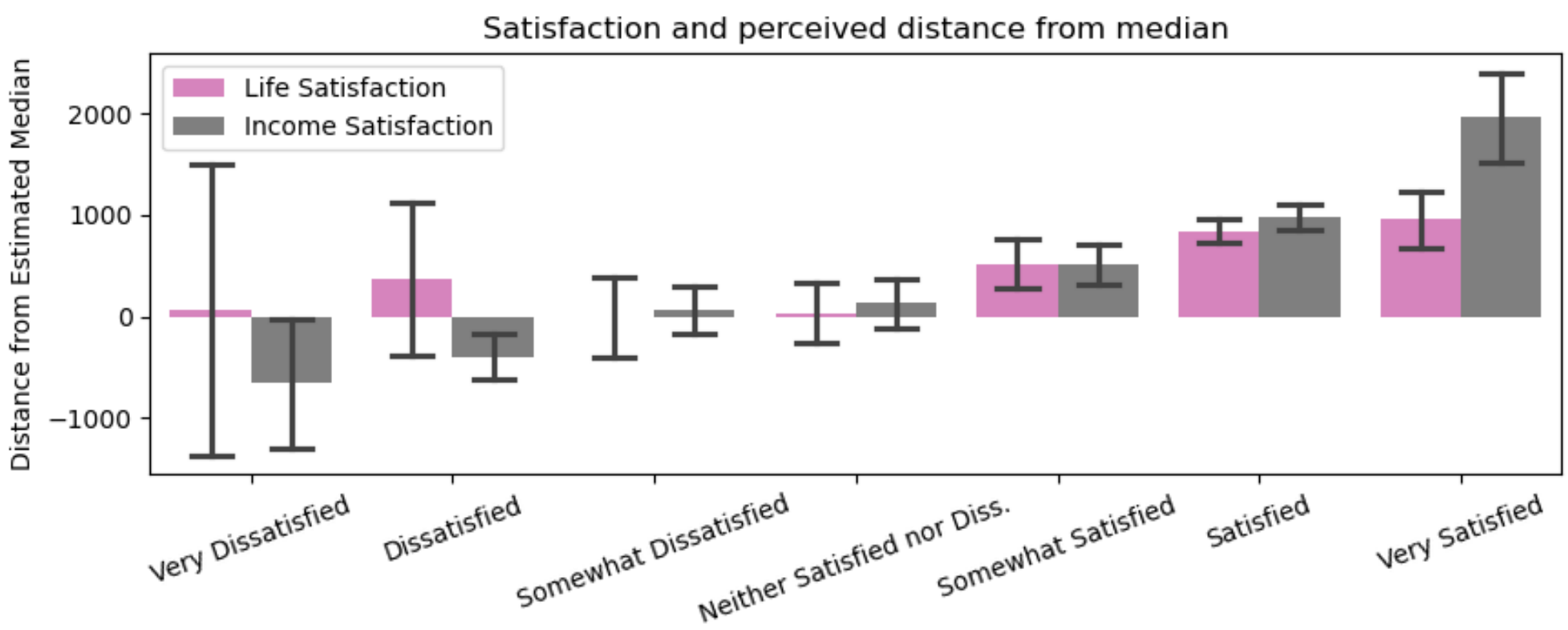

Fig. E1: **Satisfaction and subjective income comparisons.** Respondents were asked in which of seven income buckets their personal net income falls, but they were also asked to estimate the percentage of adults in the Netherlands that fall within each income bucket. We then calculate the median of these estimated distributions (using the midpoints of the buckets) and the difference with their own bucket midpoint. Answers to questions about the respondents' life and income satisfaction are on the horizontal axis. Bars indicate two times the standard error away from the mean.

The results in Figure E1 clearly indicate that the most satisfied individuals estimate themselves to be above the median on average (i.e., positive distance). This implies that when these individuals are comparing incomes, they are comparing downward, i.e., affecting their satisfaction positively, which coincides with the results of the model

(Figures 8, C1). The converse applies to respondents within the lowest satisfaction bucket. They estimate themselves to be below the median and therefore compare upward, resulting in negative effects on satisfaction.

The second survey focused on neighbourhood perceptions. It features four questions related to social comparison within the neighbourhood, and one life satisfaction question. In Figure E2 there is a clear trend in average life satisfaction: When individuals perceive that they are doing better (financially) than others in the neighbourhood, they are more satisfied. This would be the groups of respondents that (completely) agree with the statements in Figures E2b,c and (completely) disagree in Figures E2a,d. The converse also holds for downward comparisons.

The hue of the bars represents the level of agreement of the respondents with the following statement: "People in this neighbourhood share the same values" The answer is taken as a proxy for (subjective) segregation, from the perspective of the respondent, as they feel (dis)connected from the values in the neighbourhood. Conditional on the social comparison questions, when one disagrees with the values statement, life satisfaction is lower on average. These findings coincide with those of the model, the higher quantile you compare to, the more negative the long-term changes on average (Figure 8). This is even more severe for individuals that are far away from their connections in terms of income (Figure C1).

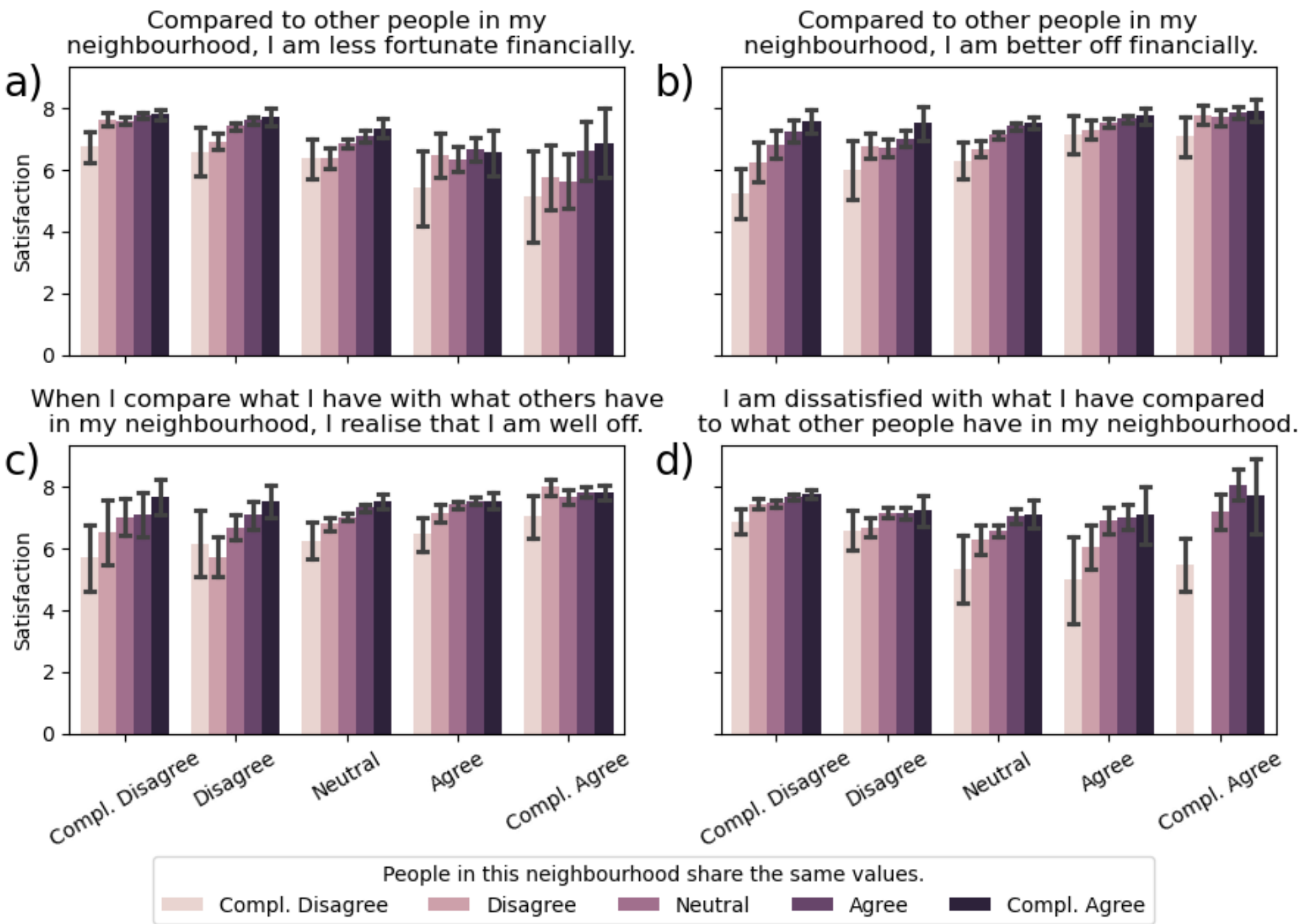

Fig. E2: **Neighbourhood Comparison.** The average life satisfaction for groups that have different levels of agreement with with the statements in the plot subtitles (bands are two standard errors from the mean). Life satisfaction is assessed by the following question: If you imagine a "ladder of life", where the first step represents the worst possible life, and the tenth (top) step the best possible life, on what step would you place yourself? Groups are further divided by their level of agreement with the following statement: People in this neighbourhood share the same values. Which is used as a proxy for segregation. Bars indicate two times the standard error away from the mean.

References

Galesic, M., Olsson, H., Rieskamp, J. (2018). A sampling model of social judgment. *Psychological review*, *125*(3), 363,

Pianosi, F., & Wagener, T. (2015). A simple and efficient method for global sensitivity analysis based on cumulative distribution functions. *Environmental Modelling & Software*, *67*, 1–11,

Pianosi, F., & Wagener, T. (2018). Distribution-based sensitivity analysis from a generic input-output sample. *Environmental Modelling & Software*, *108*, 197–207,

Scherpenzeel, A.C. (2018). ““true” longitudinal and probability-based internet panels: Evidence from the netherlands. *Social and behavioral research and the internet* (pp. 77–104). Routledge.

Tversky, A., & Kahneman, D. (1992). Advances in prospect theory: Cumulative representation of uncertainty. *Journal of Risk and uncertainty*, *5*, 297–323,
